# The Association between Dyslipidemia, Dietary Habits and Other Lifestyle Indicators among Non-Diabetic Attendees of Primary Health Care Centers in Jeddah, Saudi Arabia

**DOI:** 10.3390/nu12082441

**Published:** 2020-08-13

**Authors:** Sumia Enani, Suhad Bahijri, Manal Malibary, Hanan Jambi, Basmah Eldakhakhny, Jawaher Al-Ahmadi, Rajaa Al Raddadi, Ghada Ajabnoor, Anwar Boraie, Jaakko Tuomilehto

**Affiliations:** 1 Saudi Diabetes Research Group, King Fahd Medical Research Center, King Abdulaziz University, Jeddah 3270, Saudi Arabia; sb@kau.edu.sa (S.B.); mamalibary@kau.edu.sa (M.M.); hjambi@kau.edu.sa (H.J.); beldakhakhny@kau.edu.sa (B.E.); j_al_ahmadi@yahoo.com (J.A.-A.); rmsalharbi@kau.edu.sa (R.A.R.); gajabnour@kau.edu.sa (G.A.); boraiaa@ngha.med.sa (A.B.); jotuomilehto@gmail.com (J.T.); 2 Department of Food and Nutrition, Faculty of Human Sciences and Design, King Abdulaziz University, Jeddah 3270, Saudi Arabia; 3 Department of Clinical Biochemistry, Faculty of Medicine, King Abdulaziz University, Jeddah 22252, Saudi Arabia; 4 Department of Family Medicine, Faculty of Medicine, King Abdulaziz University, Jeddah 22252, Saudi Arabia; 5 Department of Community Medicine, Faculty of Medicine, King Abdulaziz University, Jeddah 22252, Saudi Arabia; 6 King Abdullah International Medical Research Center (KAIMRC), College of Medicine, King Saud Bin Abdulaziz, University for Health Sciences (KSAU-HS), Jeddah 22384, Saudi Arabia; 7 Department of Public Health, University of Helsinki, FI-00014 Helsinki, Finland; 8 Public Health Promotion Unit, Finnish Institute for Health and Welfare, FI-00271 Helsinki, Finland

**Keywords:** dyslipidemia, serum cholesterol, serum triglycerides, serum low density lipoprotein, serum high density lipoprotein, dietary intake, lifestyle

## Abstract

Diet and other lifestyle habits have been reported to contribute to the development of dyslipidemia in various populations. Therefore, this study investigated the association between dyslipidemia and dietary and other lifestyle practices among Saudi adults. Data were collected from adults (≥20 years) not previously diagnosed with diabetes in a cross-sectional design. Demographic, anthropometric, and clinical characteristics, as well as lifestyle and dietary habits were recorded using a predesigned questionnaire. Fasting blood samples were drawn to estimate the serum lipid profile. Out of 1385 people, 858 (62%) (491 men, 367 women) had dyslipidemia. After regression analysis to adjust for age, body mass index, and waist circumference, an intake of ≥5 cups/week of Turkish coffee, or carbonated drinks was associated with increased risk of dyslipidemia in men (OR (95% CI), 2.74 (1.53, 4.89) *p* = 0.001, and 1.53 (1.04, 2.26) *p* = 0.03 respectively), while the same intake of American coffee had a protective effect (0.53 (0.30, 0.92) *p* = 0.025). Sleep duration <6 h, and smoking were also associated with increased risk in men (1.573 (1.14, 2.18) *p* = 0.006, and 1.41 (1.00, 1.99) *p* = 0.043 respectively). In women, an increased intake of fresh vegetables was associated with increased risk (2.07 (1.09, 3.94) *p* = 0.026), which could be attributed to added salad dressing. Thus, there are sex differences in response to dietary and lifestyle practices.

## 1. Introduction

Cardiovascular diseases (CVD) are major health problems contributing to 31% of global death in 2017 according to the World Health Organization (WHO) [1]. The prevalence of CVD in Saudi Arabia in 2004 has been reported to be 5.5% [2], accounting for almost 45.7% of deaths [3]. CVD has many modifiable and non-modifiable risk factors, including certain diseases and disorders such as diabetes, hypertension, and dyslipidemia, which commonly coexist in various populations [4,5,6]. Indeed, dyslipidemia, defined as any abnormalities in serum lipids is considered atherogenic, and is reported to be associated with an increased risk of ischemic heart disease [7,8]. Several studies have been conducted to investigate the prevalence of dyslipidemia in Saudi Arabia in the past reporting an overall prevalence of 20–54% [9,10,11]. A more recent study published in 2018 on people in the eastern region of Saudi Arabia reported that the prevalence of (diagnosed and borderline) hypercholesterolemia, hypertriglyceridemia, increased low-density lipoprotein (LDL-C)-cholesterol, and decreased high density lipoprotein (HDL-C)-cholesterol were 51%, 26.9%, 38.1%, and 90.5%, respectively [12].

Many factors contribute to the development of dyslipidemia, including genetics, sex, ethnicity, increased body mass index (BMI), dietary habits, and smoking [13]. In addition, changes in sleeping patterns, and a short duration of sleep have also been associated with dyslipidemia [14]. Studies in Saudi Arabia showed that age, sex, high BMI, and waist circumference (WC), smoking, low physical activity, as well as intake of margarine were associated with dyslipidemia [10,11,12]. In spite of their reported importance in controlling dyslipidemia and CVD [15,16], the association of dietary as well as other lifestyle practices, including sleeping duration, with dyslipidemia were not fully investigated in previous Saudi studies. Saudi Arabia is a very large country, with each region having its own dietary and lifestyle characteristics. Therefore, we aimed to investigate such associations in more detail in inhabitants of the city of Jeddah, the largest city in the western region, and the gateway to the two holy cities of Islam, with a population of mixed ethnicities, bringing with them their own dietary habits. We hope that our results will help in the formulation of evidence-based dietary and lifestyle guidelines for people in our region to decrease the prevalence of dyslipidemia, which can be adopted in future, more comprehensive programs for the prevention of CVD.

## 2. Materials and Methods

### 2.1. Study Design and Sample Collection

Presented data in this study were obtained between July 2016 and February 2017 from a cross-sectional survey conducted in the city of Jeddah to develop a Saudi Dysglycemia Risk Score. The Committee on the Ethics of Human Research at the Faculty of Medicine-King Abdulaziz University, Jeddah approved the study (Reference No. 1270-13). A full explanation of the sampling methodology has been outlined in an earlier publication [17] and is summarized here as follows: adults (age ≥ 20 years), not previously diagnosed with diabetes, were recruited from attendees of primary health care centers (PHCC) in Jeddah, Saudi Arabia. An informed consent form was signed by consenting volunteers. Demographic, dietary, and lifestyle variables, as well as medical history, and family history of chronic diseases were collected from recruits using a predesigned questionnaire in the Arabic language which was based on validated questionnaires used in previous risk score studies [18,19,20,21,22] including an Arabic one [23]. The questionnaire was completed during a face to face interview by trained medical students. Anthropometric and clinical measurements (weight, height, WC, and blood pressure (BP)) were measured using standardized equipment and techniques as previously explained [17]. Weight and fat percentage were measured using a portable calibrated scale (Omron BF511; OMRON Healthcare, Kyoto, Japan). Weight and height were used to calculate BMI. A value less than 18.5 indicates underweight, while a value of 18.5–<25 kg/m^2^ indicates healthy normal weight, 25–<30 indicates overweight, and ≥30 indicates obesity. Using WC to indicate abdominal adiposity the first cut-off value for increased risk was defined as >94 cm for men, >80 cm for women, and the second cut-off value as >102 cm for men, >88 cm for women [24,25]. 

The section on dietary practices consisted of food frequency questions including 17 questions covering intake of fruits, vegetables, red meat, whole grain bread/cereals, and various commonly consumed beverages including fruit juices (fresh and otherwise), carbonated beverages, energy drinks, different types of coffee such as Arabic, American, Turkish, and cappuccino, and various teas such as red, green, cinnamon, and hibiscus tea. Participants were asked to specify whether they consumed each item more than once daily, once daily, 5–6 portions per week, 1–4 portions per week, or not at all. Food models representing portion sizes of relevant food items were presented to all participants to help in making the correct decisions. 

Fasting blood samples were taken, and serum was separated and stored at −80 °C for the estimation of the lipid profile. A flow chart outlining steps in data collection is presented in Supplementary Figure S1.

### 2.2. Biochemical Assays

Serum samples were sent regularly every 10 to 12 weeks to a collaborating laboratory at the National Guard Hospital in Jeddah which is accredited by the College of American Pathologist. Total cholesterol (TC), HDL-C and triglycerides (TG) levels were measured by spectrophotometric methods using Architect c8000 auto-analyzer (Abbott Park, IL, USA). LDL-C was calculated using the Friedewald equation [26].

### 2.3. Diagnosis of Dyslipidemia

Dyslipidemia was defined as LDL-C ≥ 3.37 mmol/L, HDL-C < 1.04 mmol/L for men and < 1.3 mmol/L for women, TC ≥ 5.18 mmol/L, TG ≥ 1.7 mmol/L or treatment with lipid-lowering drugs with all lipid levels in the normal range [27,28].

### 2.4. Statistical Analysis

IBM SPSS statistics version 20.0 for Windows was used to enter and analyze collected data. The baseline characteristics of the study population were calculated statistically and described as mean, standard deviations (SD), and frequencies. 

Demographic, lifestyle, and clinical factors of people with high levels of TC, LDL-C, and TG, and/or low level of HDL-C, as well as dyslipidemia in general, were analyzed by comparing to those with normal lipid levels. Factors with continuous variables were analyzed using an independent t-test to compare two groups, while those with categorical variables were analyzed using the Chi-square test or Fisher’s exact test, as appropriate. Unadjusted and adjusted logistic regression models were used for assessing the association between demographic, lifestyle, and dietary variables, and outcome variables: specific and general types of dyslipidemia. Stepwise regression analysis was performed to determine the dietary factors that had an influence on dyslipidemia. Independent factors included age, BMI, and WC. Only related independent variables, where *p* < 0.15 after an initial logistic regression between the dependent and independent variable, were used in the corresponding stepwise regression model. This liberal screening threshold (rather than *p* < 0.05) was used deliberately to avoid premature exclusion of potentially important covariates and to retain predictive variables with a potential or high effect, in line with established model-building guidelines [29]. This lower threshold is limited to variable inclusion only and is not a test of statistical significance.

## 3. Results

A total of 1477 adults were recruited by the end of February 2017 (Supplementary Figure S1). Complete dietary questionnaire data were obtained from 1403 people, while biochemical lipid profile data were available for 1385 people. Missing data were mainly due to missing, hemolysed, broken, or unlabeled blood samples, and there is no indication that this is related to participant characteristics or lipid status. Therefore, they are presumed to be missing at random; although this could not be directly verified, the proportion of missing data was small and unlikely to materially affect the validity of the results. Following biochemical measurements, a total of 527 people (38%) (287 men, and 240 women) were found to be normolipidemic, and 858 (62%) (491 men, and 367 women) had dyslipidemia. Thus, there was no association between sex and dyslipidemia in general. The most prevalent type of dyslipidemia was high LDL-C found in 567 people (40.9%) (341 men and 226 women), followed by high TC in 480 people (34.7%) (277 men and 203 women), and low HDL-C in 338 people (24.4%) (171 men and 167 women) and the least prevalent type of dyslipidemia was high TG levels in 301 people (21.7%) (218 men and 83 women). Therefore, increased LDL-C and TG were significantly more common in men (*p* < 0.05 and *p* < 0.001 respectively) whereas low HDL-C was significantly more common in women (*p* < 0.05).

### 3.1. Association between Dyslipidemia with Anthropometric Measurements

Demographic, clinical, and anthropometric characteristics of the study groups for men and women are presented in Supplementary Table S1. There was a significant difference in demographic and anthropometric measurements between the normolipidemic and dyslipidemic groups, with the dyslipidemia group having a significantly higher means of age, BMI, weight, body fat percentage, neck, waist and hip circumferences, and waist to hip and waist to height ratios (all *p* < 0.001, Supplementary Table S1). In addition, men and women with dyslipidemia also had significantly higher means of systolic and diastolic B*p* and significantly higher means of TC, TG, LDL-C, and lower HDL-C compared with people with normolipidemia (Supplementary Table S1). Comparing the distribution of various characteristics between the normolipidemic and the dyslipidemic groups of men and women, age, BMI, and WC were found to be significantly and directly associated with dyslipidemia (*p* < 0.001 at least, Supplementary Table S2) and all its types (*p* < 0.015 at least, Supplementary Table S2). Dyslipidemia was detected in 75% of participants over 30 years of age compared with 48.8% of those <30 years. Moreover, dyslipidemia was present in 74.8% and 72% of obese men and women compared with 53.5% and 43.7% of normal weight and 29.2% and 32% underweight men and women respectively (Supplementary Table S2a,b).

### 3.2. Patterns of Food Intake in Studied Men and Women

Recorded frequencies of the questionnaire were recategorized to no intake, 1–4 and 5 or more portions per week due to small numbers in some categories. The difference in the recorded pattern of food intake between men and women is shown in Table 1**.** Women reported eating more fresh and cooked vegetables than men, whereas men had a higher intake of red meat (*p* < 0.001 at least, Table 1). In addition, men reported drinking more fresh and non-fresh juice, soft drinks, energy drinks, red tea, and American coffee compared with women (*p* < 0.01 at least, Table 1) whereas women reported drinking more green tea, Arabic coffee, and cinnamon drink compared with men (*p* < 0.05 at least, Table 1). There were no sex-differences in the consumption of fruits, whole grain products, Turkish coffee, and hibiscus drink (Table 1).

The association between diet and dyslipidemia was analyzed in each sex group separately due to the sex differences in both the prevalence of some types of dyslipidemia as well as food intake patterns.

#### 3.2.1. Association between Food Intake and Dyslipidemia in Men

Comparing dietary habits in men between the two groups, a high intake of Turkish coffee was significantly associated with dyslipidemia (*p* < 0.001, Table 2). On the other hand, people with a moderate intake of 1–4 portions/week of American coffee had a lower prevalence of dyslipidemia (*p* = 0.027, Table 2). Dyslipidemia was detected in 79% of people drinking five or more portions a week Turkish coffee and in 65% of people who did not drink American coffee (Table 2). Turkish coffee consumption was associated in particular with low HDL-C and high TC (*p* = 0.036 and *p* = 0.018, respectively, Table 2). This association had a U-shape association as low HDL-C was detected in 22% in people with no intake, in 15% of those with an intake of 1–4 portions per week and in 29% in those with a weekly intake of five or more portions of Turkish coffee (Table 2). A similar U-shaped association was found between American coffee consumption and TC (Table 2). 

After adjusting for age, BMI, and WC the weekly intake of five or more portions of Turkish coffee was associated in men with a 2.77 increased odds for having dyslipidemia compared with those with no intake (*p* < 0.001, Table 3), while a moderate and high intake of American coffee was associated with 0.55 and 0.55 decreased odds for having dyslipidemia compared with those with no intake (*p* = 0.037 and *p* = 0.031, respectively; Table 3). After performing the multivariable regression analysis for each type of dyslipidemia separately and adjusting for age, BMI, and WC, there was no longer a significant effect of Turkish coffee on any type; however, the effect of American coffee appeared only for moderate consumption (1–4 portions/week) which was only significant for high TC (OR = 0.50; 95% CI: 0.27, 0.94; *p* = 0.038) (data not shown).

There was no significant association between the consumption of carbonated drinks and dyslipidemia when analyzed by Chi-square test. However, the regression analysis showed that the weekly intake of five or more portions of carbonated drinks was associated with a 1.56 increased odds for having dyslipidemia in general (but not specific types of dyslipidemia) in men compared with those with no intake after adjusting for age, BMI, and WC (OR = 1.53 (CI: 1.04, 2.26) *p* = 0.03, data not shown). 

There was a U-shaped association between some other dietary variables and dyslipidemia when analyzed by the Chi-square test. For example, fruits, fresh and cooked vegetables, and red tea consumption had a U-shaped relationship with low HDL-C in men. However, these food types were not predictors of dyslipidemia when analyzed using regression, adjusting for age and BMI (Table 3).

#### 3.2.2. Association between Food Intake and Dyslipidemia in Women

Comparing dietary habits in women between the two groups of normolipidemia and dyslipidemia, a high intake of the cinnamon drink was significantly associated with dyslipidemia in general (*p* = 0.027) and high LDL-C (*p* = 0.036) and TC in specific (*p* = 0.002) (Table 4). Dyslipidemia was present in 77% of women drinking five or more portions a week cinnamon drink compared to 58% in women who do not. The weekly intake of five or more portions of the cinnamon drink was associated with 2.6 times increased odds for having dyslipidemia in women compared with those with no intake. However, after adjusting for age, BMI, and WC, these odds became insignificant (Table 5). The adjustment for age and BMI regression analysis for each type of dyslipidemia separately showed that the weekly intake of five or more portions of the cinnamon drink was associated with a 2.57 times increased odds for having high TC in women compared with those with no intake (OR = 2.57; 95% CI: 1.22, 5.42; *p* = 0.02), but this was not significant after adjustment for age, BMI, and WC.

Moderate and high intakes of fresh vegetables were also found to be significantly associated with dyslipidemia (*p* < 0.001; Table 4) and mainly high LDL-C and TC (*p* = 0.034 and *p* = 0.008 respectively, Table 4). Dyslipidemia was diagnosed in 61% and 65% of women who ate either five or more portions of fresh vegetables/week or 1–4 times per week respectively compared to 38% of women who did not. This was associated with 2.50 and 2.07 times increased odds of having dyslipidemia, respectively, compared with those with no intake after adjusting for age, BMI, and WC (OR = 0.50 (95% CI: 1.29, 4.86) *p* = 0.007 and OR = 2.07 (95% CI: 1.09, 3.94) *p* = 0.026 respectively; Table 5). 

Following the finding of this interesting results, further analysis was performed to identify the characteristics of females with high intake of fresh vegetables. Women who reported to have an intake of 1–4 and 5 or more portions per week were significantly older than those who reported no intake (*p* <0.01 at least). They also had a significantly higher mean weight and neck circumference (*p* <0.05 at least). In addition, a higher proportion of these women were previously diagnosed with dyslipidemia and taking medication, however, this was not statistically significant (Supplementary Table S3).

There was no effect of other food types on dyslipidemia in women when analyzed using regression analysis after adjusting for age, BMI, and WC.

### 3.3. Association between Lifestyle Characteristics and Dyslipidemia in Men and Women

After analyzing sex groups separately, only short and long sleep durations were associated with general dyslipidemia in men only (Supplementary Table S2a, *p* = 0.012). Dyslipidemia was detected in 68.5% and 68.2% of men who reported sleeping either <6 h/day, or >8 h/day respectively compared with 58.1% of people who slept 6–8 h per day. This association was particularly evident for increased TC (*p* < 0.029). There was no association between sleep duration and dyslipidemia in women.

There was no association between physical activity or daily sitting duration and dyslipidemia in general (Supplementary Table S2a,b). However, low physical activity was associated with high TC and TG in men (*p* = 0.024 and *p* = 0.048 respectively; Supplementary Table S2a). High TC and TG were detected in 39.1% and 30.9% of men reporting low physical activity compared with 31.3% and 24.5%, respectively, in men who were more physically active. In women, low physical activity was associated with low HDL-C only (*p* = 0.024; Supplementary Table S2b). Low HDL-C was detected in 41% of women reporting low physical activity compared with 32% of women who were more physically active. On the other hand, very short sitting duration (<4 h per day) was associated with decreased HDL-C in women as it was found in 36.3% of women reporting sitting for <4 h per day compared with 20–26% of those sitting for more than 4 h (*p* = 0.031, Supplementary Table S2b).

Smoking was not associated with dyslipidemia in general (Supplementary Table S2a,b). However, it was associated with high TG in men (*p* = 0.011; Supplementary Table S2a). High TG was detected in 34.2% of men who smoke compared with 25.7% of non-smoking men. In women, smoking was associated with low HDL-C (*p* = 0.035; Supplementary Table S2b). Low HDL-C was detected in 50% of women who smoked compared with 36.1% of who were non-smokers.

After adjusting for age, BMI, and WC, only sleep duration and smoking were predictors for dyslipidemia in men (Table 3). Having <6 h of sleep per day was associated with increased odds of having general dyslipidemia compared with those sleeping 6–8 h (OR = 1.57 (CI: 1.14, 2.18) *p* = 0.006; Table 3). In addition, smoking was associated with increased odds of having general dyslipidemia compared with those who did not smoke (OR = 1.42 (CI: 1.00, 1.98) *p* = 0.043; Table 3).

Lifestyle characteristics were not predictors for dyslipidemia in women after adjusting for age, BMI, and WC.

## 4. Discussion

In this study, we investigated the association between dyslipidemia, dietary, and other lifestyle practices among Saudi adults not previously diagnosed with diabetes. Dyslipidemia was found to be associated with increased age, BMI, and WC in men and women. However, notable differences between sexes in the association of dyslipidemia with dietary and other lifestyle practices were noted. In men, an increased risk of dyslipidemia was found to be associated with high consumption of Turkish coffee, and carbonated drinks, short (<6 h) and long (>8 h) sleep duration, and smoking, while high consumption of American coffee was associated with decreased risk after adjusting for age, BMI and WC. However, only increased consumption of fresh vegetables was associated with an increased risk of dyslipidemia in women after adjusting for confounding factors.

The prevalence of dyslipidemia among Saudi adults in our study was 62% which is higher than previously reported [10,11]. The difference could be due to the variations in dietary, and lifestyle practices between regions of the Kingdom as well ethnic origin of studied populations since ethnicity among Saudi nationals can vary to some extent and influence genetic and lifestyle factors. Our study was conducted in the city of Jeddah in the Western Region of the Kingdom, while the previous study [10,11] included people from all the regions in Saudi Arabia. Another reason could be the variation in defining dyslipidemia and all its types. In this study, the used dyslipidemia definition was more comprehensive as it included laboratory-measured high TG, high fasting serum cholesterol, low fasting plasma HDL-C and/or high fasting plasma LDL-C levels, as well as treatment with hyperlipidemic drugs [27,28].

The most prevalent type of dyslipidemia in this study was high LDL-C which was detected in 40.9% of studied Saudi adults. This was followed by high TC in 34.7%, low HDL-C in 24.4%, and least of all high TG levels in 21.7% of the study population. A comparably high prevalence of high LDL-C and hypercholesterolemia in a Saudi population was reported in previous large community-based national cross-sectional studies [9,10,11]. However, one of these studies [9] reported a much higher prevalence for hypertriglyceridemia despite using similar definition. This could be caused by the difference in the age range of participants as the previous study [9] included people aged 30–70 years, whereas the current study included adults ≥18 years of age.

As reported in previous studies on Saudi and other populations [9,10,11,30,31] older age, increased BMI and WC were associated with a higher prevalence of dyslipidemia in general, and all its types. The prevalence of high TG in the current study was lower than previously reported [11] being 11.4% for young adults aged 30 years or under compared to with 32% in the older adults aged more than 30 years. This difference in prevalence is due to the variation in the cut-off point to determine hypertriglyceridemia as it was ≥ 1.7 mmol/L in our study as well as in that of Al-Amri et al. [32] whereas ≥ 1.27 mmol/L was adopted in the previous study [11]. The cut-off point in this previous study allowed a wider range of the population to be diagnosed with hypertriglyceridemia, and therefore a higher prevalence was reported.

In our study sex had no effect on the prevalence of dyslipidemia in general but both hypertriglyceridemia and high LDL-C were more prevalent in men than women. Similar sex-differences were reported previously [9,11]. This could be due to the higher proportion of smokers, and the higher prevalence of low sleep duration (<6 h) among men. These lifestyle factors were associated with both hypertriglyceridemia and elevated LDL-C in this study. On the other hand, low HDL-C was more prevalent among women, which is different from that previously reported [11]. Similar sex differences in the prevalence of low HDL-C was previously found in Iranian population [33], which reported a much higher prevalence in both men and women compared with that found in our study. 

Emerging evidence collected from self-reported data indicated that both short and long sleep duration are associated with adverse health consequences including obesity hypertension, diabetes mellitus, and CVDs, showing a curvilinear relationship between habitual sleep duration and these conditions [34,35,36]. In line with this, in our study in men but not in women, both short (<6 h) and long (>8 h) sleep durations were associated with dyslipidemia, particularly with elevated LDL-C and TG levels. In contrast, a recent systemic review, meta-analysis, and meta-regression suggested that a causal relationship existed between short sleep durations and adverse health outcomes including increased risk of diabetes, hypertension, and CVD [14]. However, there was insufficient data to draw a conclusion regarding the association between short sleep duration and dyslipidemia [14,34]. On the other hand, another systematic review indicated that long sleep may have more adverse health effect than short sleep [37]. Findings from cross-sectional data for the middle-aged and older Chinese population partly supported our finding that both short and long sleep durations were associated with dyslipidemia; since their cohort data suggested that long sleep duration (>9 h) only was associated with dyslipidemia [35].

In experimental studies, sleep debt has shown to induce alterations in the endocrine functions, so that sleep debt for 4 h resulted in raising evening cortisol concentrations, suggesting that it results in the disturbance of the negative-feedback regulation of the hypothalamus-pituitary-adrenal axis [37,38]. A previous cross-sectional study reported that excess cortisol secretion was linked to increased TG concentrations in South Asians [39]. The association between sleep duration, cortisol secretion, and dyslipidemia needs to be further investigated [40]. Even though the cortisol level was not measured in our study, dysregulated cortisol secretion might be the cause for the observed association between short sleep duration and dyslipidemia. 

High consumption of Turkish coffee in this study was associated with dyslipidemia in men only. This is consistent with findings from previous clinical studies [41]. In contrast, we found that high consumption of American coffee was associated with decreased risk of dyslipidemia in men also, which contradicts findings in a clinical cohort study reporting that abstention from filtered coffee for 3 weeks resulted in a decrease in TC [42]. In an attempt to explain the different findings, a previous review suggested that brewing and preparation techniques influence this association as the long contact of beans with hot water in unfiltered coffee results in the formation of cholesterol elevating compounds, such as cafestol and kahweol, and that up to 90% of these diterpenes may be carried by floating coffee bean particles [43]. The same review suggested that the predicted rise in serum cholesterol levels with consumption of five cups of Turkish coffee per day is 0.25 mmol/L whereas filtered coffee is not predicted to increase serum cholesterol levels. In addition to diterpene alcohols, other compounds are found in the complex coffee beverage [43]. Moreover, a previous meta-regression analysis revealed that coffee consumption, particularly unfiltered coffee, is related to increased LDL-C, TC, and TG in a dose dose-dependent manner [42]. Coffee also contains antioxidant and anti-inflammatory compounds such as chlorogenic acid [43]. This might explain the observed U-shaped relationship between coffee consumption and low HDL-C and high TC in the current study. The observation that Turkish coffee was related to increased dyslipidemia in men but not women in the current study cannot be explained by variation in consumption patterns between sexes since these were comparable. Similar to our findings, a previous Norwegian study reported that unfiltered coffee consumption was associated with elevated cholesterol levels in men but not women [44]. 

In the current study fruit and vegetable intake were associated with dyslipidemia in men in a U-shaped manner. However, following adjustment for age, BMI, and WC consumption of fruits and vegetables lost their association with dyslipidemia in men. This suggests that this association could be caused by the high intake of total food in general and thus confounded by other constituents of diet. On the other hand, the consumption of fruit and fresh vegetables was associated with an increased risk of dyslipidemia, particularly elevated TC, in women, thus contradicting the previously reported beneficial effects of fruits and vegetables on serum lipids [45]. However, following adjustment for age, BMI, and WC, only the consumption of fresh vegetables retained its association with dyslipidemia. This could be a chance finding given several statistical comparisons, or due to the residual confounding since we did not collect data on all possible factors that may be associated with dyslipidemia. Following this unexpected finding, further statistical analysis was carried out, and the mean neck circumference (NC) was found to be higher in women reporting medium and high intake of fresh vegetables. In a systemic review and meta-analysis, positive associations between NC, high TC, and LDL-C, and low HDL-C concentrations were found, and subjects with higher NC had approximately two-fold higher risk for hypertriglyceridemia compared to those with lower NC [46]. NC was not adjusted for in our study, hence its increased mean in women consuming fresh vegetables could contribute to the noted association. Another reason could be that women who knew about their dyslipidemia or were trying to lose weight had modified their diet to include more fresh vegetables, i.e., representing reverse causation. Indeed, the mean weight of women ingesting fresh vegetables was significantly higher than the mean of those reporting no intake, and a higher percentage were on lipid-lowering therapy, but the difference to those reporting no intake did not reach statistical significance. Also, the addition of salad dressings to fresh vegetable salads is a very common dietary practice. Salad dressings often contain around 40% of fat, and a considerable amount of high fructose corn syrup, which has been proven to increase lipid synthesis and is associated with dyslipidemia [47]. Furthermore, mayonnaise is a major ingredient as a salad dressing alone or as part of other popular dressings (e.g., thousand islands), and studies have found that the use of palm oil, a cheaper oil comparatively, in its preparation caused an increase in total and LDL-C compared to the use of soybean oil, which is a more expensive type [48]. Therefore, the consumption of dressing can distort potential beneficial influences of vegetable consumption on serum lipids.

Studies with more detailed dietary assessment methods such as food diaries that provide information about consumed additives to vegetables are required to get a clear picture of the influence of vegetable consumption on serum lipids in Saudis. 

It was interesting to note that in men, smoking and high consumption of carbonated drinks were significantly associated with dyslipidemia only following adjustment for age, BMI, and WC. Both cigarette smoking and dyslipidemia are well-established major risk factors for cardiovascular disease. Studies on different populations reported an increased risk of dyslipidemia in smokers [1,2,3]. A Korean cross-sectional study [49] conducted in adults aged ≥19 years reported that there was an increased risk of low HDL-C, high TG and high LDL-C in male smokers compared with non-smokers. On the other hand, female smokers were found to have a significantly increased risk for high TC, high TG, and high LDL-C compared with non-smokers. These results emphasize the sex difference in response to the same lifestyle factors as noted in our study. A Tunisian study [50] reported an increased risk of high triglyceridemia, and low HDL-C in smokers in a dose-response manner. Our study did not investigate the amount or type of smoking, however it was noted that a higher percentage of smokers had hypertriglyceridemia. Similar findings were reported in a Chinese study of elderly adults [51] that concluded that smoking was an independent risk factor for dyslipidemia and that it had a bigger effect than other studied factors including alcohol intake, BMI, and age. 

High consumption of carbonated drinks was a predictor of dyslipidemia in men. A prospective study reported that daily consumption of carbonated drinks was associated with increased risk of hypertriglyceridemia and low HDL-C. In a cross-sectional study performed in Oslo, Norway, the consumption of colas but not other carbonated drinks was associated with low serum HDL-C, as well as high TG and LDL-C [52]. The association between high carbonated drink intake and dyslipidemia could be attributed to the high sucrose content in these drinks as it was reported to be linked to hypertriglyceridemia previously [53]. The questionnaire that was used in this study did not provide specific data about the type of carbonated drink. Future work should include more details on type of drinks, and whether it was sugar-free or not.

A number of lifestyle practices showed an association initially, but this association was lost after adjustment for confounding factors. These practices include decreased physical activity and sitting duration. Physical activity and sedentary behavior are common factors reported to be associated with serum lipid levels [54,55]. A previous review on the effect of aerobic exercise on serum lipids recommended that clinicians should rely more on physical activity and less on lipid lowering drugs to modify lipid variables, thus to reduce the risk of myocardial infractions and CVD [56]. Low physical activity was found to be associated with dyslipidemia and particularly with high TC and TG in men, but it was not a significant predictive factor. However, the questionnaire used in our study did not provide detailed information about the type, timing, and intensity of physical activity. People tend to start exercising in an attempt to control weight, and various ill-health conditions, which could explain the loss of association following adjustment for BMI and WC in our study. Specific specialized questionnaires should be used in future work to provide more detailed information about physical activity, which would aid in providing more information about the association between physical activity and dyslipidemia in the Saudi population.

Very short sitting duration (for less than 4 h) was associated with a decreased level of HDL-C in women in the current study. Short sitting duration might be associated with stress at work. Indeed, it has been observed in individuals whose career requires high labor work and low sitting hours and provides low salaries [57]. A previous cross-sectional study on women employees of a retail company in Japan reported that employees with effort-reward imbalance had a 4.4-fold higher risk of low HDL-C compared with employees who have balanced effort-reward [57]. This suggests that the stress in careers or other work-related factors could distort the relationship between physical activity and dyslipidemia and might explain the observed relationship between low sitting duration and unfavorable HDL-C profile noted in our study. 

Another unexpected finding in this study was that cinnamon drink consumption was associated with increased risk of dyslipidemia, particularly elevated TC, in women before adjustment for confounding factors. This was not observed in men probably due to the higher intake of cinnamon drink in women. In contrast, a previous systematic review and meta-analysis suggested that cinnamon supplementation has an anti-lipidemic effect especially on plasma cholesterol and TG in a duration-dependent but not dose-dependent manner [58]. After adjusting for age, BMI, and WC the association ceased to exist in our study. The inverse association between cinnamon drink consumption and dyslipidemia observed might be due to the addition of sugar to the drink which is a common practice among Saudis. Another possible explanation could be due to changes in the diet by individuals who have been recently diagnosed with dyslipidemia or hyperglycemia. The current study had a cross-sectional design, which is the main limitation since it did not provide information on the duration of the recorded dietary pattern. Cinnamon drink is commonly ingested by Saudi individuals who are diagnosed with dysglycemia due to the common belief that cinnamon lowers blood sugar. Since dysglycemia occurs more in individuals with dyslipidemia particularly high LDL-C [32], changes in dietary habits such as increasing consumption of cinnamon drink or fresh vegetables in individuals diagnosed with dysglycemia or dyslipidemia can result in biased observations regarding the effect of these food types on serum lipids. In order to obtain a clear picture, future studies should include information on the duration of the recorded dietary habits, and whether dietary practices have been changed due to medical advice.

There are limitations to our study. First, its cross-sectional design did not allow for inferences about cause and effect and can suggest associations only. In addition, collected data was based on a questionnaire providing self-reported dietary and lifestyle data, hence errors of reporting are expected. However, conducting face to face interviews by trained data collectors is believed to minimize such errors. In addition, our study had a relatively small sample size, but it was enough to detect several associations. Given the large number of dietary, lifestyle, and lipid variables examined, numerous statistical comparisons were performed. No formal adjustments were made for these multiple comparisons. Therefore, the associations reported here should be considered exploratory and hypothesis-generating, not confirmatory, and some results may represent random associations. These findings require confirmation in adequately powered, prospectively designed studies.

In spite of the limitation, our study has many points of strength. The first being that bias was avoided by a random selection of PHC and included volunteers. Secondly, standardized methods for data collections by well-trained data collectors were used. Moreover, a comprehensive definition was used to diagnose dyslipidemia, and all collected samples were analyzed in one accredited lab to avoid variations leading to misclassification.

## 5. Conclusions

In this studied population, there was a high prevalence of dyslipidemia among men and women which was found to be associated with increased age, BMI, and WC. Dyslipidemia was associated with several lifestyle and dietary factors, which was sex-specific. After adjusting for age, BMI, and WC, short (<6 h), and long (>8 h) sleep duration, and high consumption of Turkish coffee and carbonated drinks were associated with increased risk of dyslipidemia in men, while high consumption of American coffee was associated with a decreased risk. However, in women, only increased consumption of fresh vegetables was associated with an increased risk of dyslipidemia after adjusting for confounding factors.

This highlights the necessity of the adjustment of these modifiable risk factors since individuals with dyslipidemia are at increased risk of developing CVD. More detailed cohort studies are needed to reach firmer conclusions and lead to prevention recommendations. Nevertheless, results from this study provide useful information for the planning of future preventive actions against CVD.

## Figures and Tables

**Table 1:** Food intake pattern by sex.

	Men	Women	χ^2^ (*p*-Value)
	N (N%)	N (N%)	
Fruit (portion)			
No intake	111 (14.0%)	72 (11.8%)	1.727 *p* = 0.422
1–4/week	399 (50.4%)	323 (52.8%)	
5 or more/week	281 (35.5%)	217 (35.5%)	
Fresh vegetable (portion)			
No intake	115 (14.5%)	53 (8.7%)	**15.259** ***p* < 0.001**
1–4/week	310 (39.2%)	225 (36.8%)	
5 or more/week	366 (46.3%)	334 (54.6%)	
Cooked vegetable (portion)			
No intake	164 (20.7%)	67 (10.9%)	**27.975** ***p* < 0.001**
1–4/week	356 (45.0%)	278 (45.4%)	
5 or more/week	271 (34.3%)	267 (43.6%)	
Whole grains (portion)			
No intake	113 (14.3%)	93 (15.2%)	3.187 *p* = 0.203
1–4/week	146 (18.5%)	91 (14.9%)	
5 or more/week	532 (67.3%)	428 (69.9%)	
Red meat (portion)			
No intake	58 (7.3%)	139 (22.7%)	**77.64** ***p* < 0.001**
1–4/week	453 (57.3%)	335 (54.7%)	
5 or more/week	280 (35.4%)	138 (22.5%)	
Fresh juice (portion)			
No intake	182 (23.0%)	186 (30.4%)	**10.039** ***p* = 0.007**
1–4/week	412 (52.1%)	281 (45.9%)	
5 or more/week	197 (24.9%)	145 (23.7%)	
Non fresh juice (portion)			
No intake	252 (31.9%)	259 (42.3%)	**20.572** ***p* < 0.001**
1–4/week	253 (32.0%)	191 (31.2%)	
5 or more/week	286 (36.2%)	162 (26.5%)	
Soft drinks (portion)			
No intake	247 (31.2%)	298 (48.7%)	**56.848** ***p* < 0.001**
1–4/week	233 (29.5%)	175 (28.6%)	
5 or more/week	311 (39.3%)	139 (22.7%)	
Red tea (portion)			
No intake	166 (21.0%)	182 (29.7%)	**14.346** ***p* = 0.001**
1–4/week	201 (25.4%)	133 (21.7%)	
5 or more/week	424 (53.6%)	297 (48.5%)	
Green tea (portion)			
No intake	464 (58.7%)	323 (52.8%)	**7.39** ***p* = 0.025**
1–4/week	168 (21.2%)	130 (21.2%)	
5 or more/week	159 (20.1%)	159 (26.0%)	
Arabic coffee (portion)			
No intake	347 (43.9%)	232 (37.9%)	**10.33** ***p* = 0.006**
1–4/week	214 (27.1%)	153 (25.0%)	
5 or more/week	230 (29.1%)	227 (37.1%)	
Turkish coffee (portion)			
No intake	565 (71.4%)	440 (71.9%)	0.112 *p* = 0.946
1–4/week	124 (15.7%)	92 (15.0%)	
5 or more/week	102 (12.9%)	80 (13.1%)	
American coffee (portion)			
No intake	626 (79.1%)	531 (86.8%)	**14.183** ***p* = 0.001**
1–4/week	81 (10.2%)	43 (7.0%)	
5 or more/week	84 (10.6%)	38 (6.2%)	
Cappuccino (portion)			
No intake	536 (67.8%)	368 (60.1%)	**9.881** ***p* = 0.007**
1–4/week	157 (19.8%)	139 (22.7%)	
5 or more/week	98 (12.4%)	105 (17.2%)	
Energy drinks (portion)			
No intake	623 (78.8%)	545 (89.1%)	**26.237** ***p* < 0.001**
1–4/week	115 (14.5%)	45 (7.4%)	
5 or more/week	53 (6.7%)	22 (3.6%)	
Hibiscus drinks (portion)			
No intake	719 (90.9%)	556 (90.8%)	0.57 *p* = 0.752
1–4/week	58 (7.3%)	42 (6.9%)	
5 or more/week	14 (1.8%)	14 (2.3%)	
Cinnamon drink (portion)			
No intake	707 (89.4%)	478 (78.1%)	**37.241** ***p* < 0.001**
1–4/week	52 (6.6%)	100 (16.3%)	
5 or more/week	32 (4.0%)	34 (5.6%)	

Data is shown as frequencies and percentages of all people in the sex group. χ^2^ is the chi-square test value followed by its *p*-value. Significant differences between groups are shown in bold font. This table is based on participants with completed dietary questionnaire data (*n* = 1403). Analyses involving lipid outcomes are restricted to participants with available biochemical lipid profile data (*n* = 1385).

**Table 2:** Comparison of dietary habits between normolipidemic and dyslipidemic men groups presented as number of men (%) for overall dyslipidemia, and abnormalities in different lipid parameters.

Variable	General Dyslipidemia		LDL-C		HDL-C		TC		TG	
	No (*n* = 287)	Yes (*n* = 491)	Normal (*n* = 437)	High (*n* = 341)	Normal (*n* = 607)	Low (*n* = 171)	Normal (*n* = 501)	High (*n* = 277)	Normal (*n* = 560)	High (*n* = 218)
	N (%)	N (%)	N (%)	N (%)	N (%)	N (%)	N (%)	N (%)	N (%)	N (%)
Fruit (portion)										
No intake	39 (35.8)	70 (64.2)	60 (55.0)	49 (45.0)	84 (77.1)	25 (22.9)	66 (60.6)	43 (39.4)	71 (65.1)	38 (34.9)
1–4/week	154 (39.1)	240 (60.9)	217 (55.1)	177 (44.9)	328 (83.2)	66 (16.8)	256 (65.0)	138 (35.0)	290 (73.6)	104 (26.4)
5 or more/week	94 (34.2)	181 (65.8)	160 (58.2)	115 (41.8)	195 (70.9)	80 (29.1)	179 (65.1)	96 (34.9)	199 (72.4)	76 (27.6)
X^2^ (*p*-value)	1.74 (*p* = 0.419)		0.7 (*p* = 0.705)		**14.448 (*p* = 0.001)**		0.818 (*p* = 0.664)		3.066 (*p* = 0.216)	
Fresh vegetable (portion)										
No intake	43 (37.7)	71 (62.3)	68 (59.6)	46 (40.4)	91 (79.8)	23 (20.2)	72 (63.2)	42 (36.8)	78 (68.4)	36 (31.6)
1–4/week	120 (39.3)	185 (60.7)	160 (52.5)	145 (47.5)	254 (83.3)	51 (16.7)	195 (63.9)	110 (36.1)	236 (77.4)	69 (22.6)
5 or more/week	124 (34.5)	235 (65.5)	209 (58.2)	150 (41.8)	262 (73.0)	97 (27.0)	234 (65.2)	125 (34.8)	246 (68.5)	113 (31.5)
X^2^ (*p*-value)	1.674 (*p* = 0.433)		2.878 (*p* = 0.237)		**10.452 (*p* = 0.005)**		0.201 (*p* = 0.905)		**7.247 (*p* = 0.027)**	
Cooked vegetable (portion)										
No intake	66 (41.3)	94 (58.8)	97 (60.6)	63 (39.4)	128 (80.0)	32 (20.0)	111 (69.4)	49 (30.6)	126 (78.8)	34 (21.3)
1–4/week	128 (36.4)	224 (63.4)	187 (53.1)	165 (46.9)	291 (82.7)	61 (17.3)	212 (60.2)	140 (39.8)	245 (69.6)	107 (30.4)
5 or more/week	93 (35.8)	173 (65.0)	153 (57.5)	113 (42.5)	188 (70.7)	78 (29.3)	178 (66.9)	88 (33.1)	189 (71.1)	77 (28.9)
X^2^ (*p*-value)	1.773 (*p* = 0.412)		2.812 (*p* = 0.245)		**13.17 (*p* = 0.001)**		5.136 (*p* = 0.077)		4.736 (*p* = 0.077)	
Whole grains (portion)										
No intake	40 (36.4)	70 (63.6)	64 (58.2)	46 (41.8)	81 (73.6)	29 (26.4)	69 (62.7)	41 (37.3)	79 (71.8)	31 (28.2)
1–4/week	54 (37.5)	90 (62.5)	77 (53.5)	67 (46.5)	118 (81.9)	26 (18.1)	8(61.1)	56 (38.9)	105 (72.9)	39 (27.1)
5 or more/week	193 (36.8)	331 (63.2)	296 (56.5)	228 (43.5)	408 (77.9)	116 (22.1)	344 (65.6)	180 (34.4)	376 (71.8)	148 (28.2)
X^2^ (*p*-value)	0.037 (*p* = 0.982)		0.628 (*p* = 0.73)		2.533 (*p* = 0.282)		1.17 (*p* = 0.557)		0.77 (*p* = 0.962)	
Red meat (portion)										
No intake	19 (33.3)	38 (66.7)	33 (57.9)	24 (42.1)	41 (71.9)	16 (28.1)	35 (61.4)	22 (38.6)	43 (75.4)	14 (24.6)
1–4/week	167 (37.5)	287 (62.5)	260 (58.4)	185 (41.6)	347 (78.0)	98 (22.0)	290 (65.2)	155 (34.8)	317 (71.2)	128 (28.8)
5 or more/week	101 (36.6)	175 (63.4)	144 (52.2)	132 (47.8)	219 (79.3)	57 (20.7)	176 (63.8)	100 (36.2)	200 (72.5)	76 (27.5)
X^2^ (*p*-value)	0.398 (*p* = 0.82)		2.78 (*p* = 0.249)		1.517 (*p* = 0.468)		0.386 (*p* = 0.825)		0.492 (*p* = 0.782)	
Fresh juice (portion)										
No intake	72 (40.2)	107 (59.8)	112 (62.6)	67 (37.4)	133 (74.3)	46 (25.7)	124 (69.3)	55 (30.7)	128 (71.5)	51 (28.5)
1–4/week	141 (34.8)	264 (65.2)	213 (52.6)	192 (47.4)	319 (78.8)	86 (21.2)	249 (61.5)	156 (38.5)	294 (72.6)	111 (27.4)
5 or more/week	74 (38.1)	120 (61.9)	112 (57.7)	82 (42.3)	155 (79.9)	39 (20.1)	128 (66.0)	66 (34.0)	138 (71.1)	56 (28.9)
X^2^ (*p*-value)	1.735 (*p* = 0.42)		5.275 (*p* = 0.072)		1.973 (*p* = 0.373)		3.57 (*p* = 0.168)		0.164 (*p* = 0.921)	
Non fresh juice (portion)										
No intake	87 (34.9)	162 (65.1)	141 (56.6)	108 (43.4)	186 (74.7)	63 (25.3)	155 (62.2)	94 (37.8)	179 (71.9	70 (28.1)
1–4/week	97 (38.8)	153 (61.2)	136 (54.4)	114 (45.6)	200 (80.0)	50 (20.0)	158 (63.2)	92 (36.8)	183 (73.2)	67 (26.8)
5 or more/week	103 (36.9)	176 (63.1)	160 (57.3)	119 (42.7)	221 (79.2)	58 (20.8)	188 (67.4)	91 (32.6)	198 (71.0)	81 (29.0)
X^2^ (*p*-value)	0.779 (*p* = 0.671)		0.496 (*p* = 0.78)		2.404 (*p* = 0.301)		1.743 (*p* = 0.418)		0.327 (*p* = 0.849)	
Carbonated drinks (portion)										
No intake	92 (38.2)	149 (61.8)	145 (60.2)	96 (39.8)	184 (76.3)	57 (23.7)	152 (63.1)	89 (36.9)	173 (71.8)	68 (28.2)
1–4/week	88 (37.9)	144 (62.1)	126 (54.3)	106 (45.7)	187 (80.6)	45 (19.4)	155 (66.8)	77 (33.2)	171 (73.7)	61 (26.3)
5 or more/week	107 (35.1)	198 (64.9)	166 (54.4)	139 (45.6)	236 (77.4)	69 (22.6)	194 (63.6)	111 (36.4)	216 (70.8)	89 (29.2)
X^2^ (*p*-value)	0.707 (*p* = 0.702)		2.266 (*p* = 0.322)		1.369 (*p* = 0.504)		0.857 (*p* = 0.615)		0.551 (*p* = 0.759)	
Red tea (portion)										
No intake	61 (37.2)	103 (62.8)	98 (59.8)	66 (40.2)	130 (79.3)	34 (20.7)	112 (68.3)	52 (31.7)	124 (75.6)	40 (24.4)
1–4/week	82 (41.6)	115 (58.4)	110 (55.8)	87 (44.2)	168 (85.3)	29 (14.7)	131 (66.5)	66 (33.5)	149 (75.6)	48 (24.4)
5 or more/week	144 (34.5)	273 (65.5)	229 (54.9)	188 (45.1)	309 (74.1)	108 (25.9)	258 (61.9)	159 (38.1)	287 (68.8)	130 (31.2)
X^2^ (*p*-value)	2.899 (*p* = 0.235)		1.132 (*p* = 0568)		**9.938 (*p* = 0.007)**		2.626 (*p* = 0.269)		4.434 (*p* = 0.109)	
Green tea (portion)										
No intake	174 (38.2)	281 (61.8)	263 (57.8)	192 (42.2)	354 (77.8)	101 (22.2)	304 (66.8)	151 (33.2)	329 (72.3)	126 (27.7)
1–4/week	62 (37.6)	103 (62.4)	87 (52.7)	78 (47.3)	135 (81.8)	30 (18.2)	98 (59.4)	67 (40.6)	123 (74.5)	42 (25.5)
5 or more/week	51 (32.3)	107 (67.7)	87 (55.1)	71 (44.9)	118 (74.7)	40 (25.3)	99 (62.7)	59 (37.3)	108 (68.4)	50 (31.6)
X^2^ (*p*-value)	1.834 (*p* = 0.4)		1.365 (*p* = 0.505)		2.426 (*p* = 0.297)		3.168 (*p* = 0.205)		1.592 (*p* = 0.451)	
Arabic coffee (portion)										
No intake	124 (36.3)	218 (63.7)	197 (57.6)	145 (42.4)	261 (76.3)	81 (23.7)	218 (63.7)	124 (36.3)	236 (69.0)	106 (31.0)
1–4/week	84 (40.0)	126 (60.0)	112 (53.3)	98 (46.7)	176 (83.8)	34 (16.2)	139 (66.2)	71 (33.8)	163 (77.6)	47 (22.4)
5 or more/week	79 (35.0)	147 (65.0)	128 (56.6)	98 (43.4)	170 (75.2)	56 (24.8)	144 (63.7)	82 (36.3)	161 (71.2)	65 (28.8)
X^2^ (*p*-value)	1.294 (*p* = 0.524)		0.991 (*p* = 0.609)		5.716 (*p* = 0.057)		0.404 (*p* = 0.817)		4.872 (*p* = 0.087)	
Turkish coffee (portion)										
No intake	212 (38.0)	346 (62.0)	317 (56.8)	241 (43.2)	434 (77.8)	124 (22.2)	361 (64.7)	197 (35.3)	401 (71.9)	157 (28.1)
1–4/week	54 (44.6)	67 (55.4)	72 (59.5)	49 (40.5)	103 (85.1)	18 (14.9)	87 (71.9)	34 (28.1)	94 (77.7)	27 (22.3)
5 or more/week	21 (21.2)	78 (78.8)	48 (48.5)	51 (51.5)	70 (70.7)	29 (29.3)	53 (53.5)	46 (46.5)	65 (65.7)	34 (34.3)
X^2^ (*p*-value)	**13.856** **(*p* ≤0.001)**		3.014 (*p* = 0.222)		**6.667 (*p* = 0.036)**		**8.087 (*p* = 0.018)**		3.92 (*p* = 0.141)	
American coffee (portion)										
No intake	215 (34.8)	402 (65.2)	341 (55.3)	276 (44.7)	472 (76.5)	145 (23.5)	393 (63.7)	224 (36.3)	435 (70.5)	182 (29.5)
1–4/week	40 (50.0)	40 (50.0)	53 (66.3)	27 (33.8)	69 (86.3)	11 (13.8)	63 (78.8)	17 (21.3)	64 (80.0)	16 (20.0)
5 or more/week	32 (39.5)	49 (60.5)	43 (53.1)	38 (46.9)	66 (81.5)	15 (18.5)	45 (55.6)	36 (44.4)	61 (75.3)	20 (24.7)
X^2^ (*p*-value)	**7.251** **(*p* = 0.027)**		3.819 (*p* = 0.148)		4.558 (*p* = 0.102)		**10.082 (*p* = 0.006)**		3.664 (*p* = 0.16)	
Cappuccino (portion)										
No intake	195 (36.9)	333 (63.1)	298 (56.4)	230 (43.6)	404 (76.5)	124 (23.5)	342 (64.8)	186 (35.2)	380 (72.0)	148 (28.0)
1–4/week	64 (41.6)	90 (58.4)	88 (57.1)	66 (42.9)	130 (84.4)	24 (15.6)	104 (67.5)	50 (32.5)	114 (74.0)	40 (26.0)
5 or more/week	28 (29.2)	68 (70.8)	51 (53.1)	45 (46.9)	73 (76.0)	23 (24.0)	55 (57.3)	41 (42.7)	66 (68.8)	30 (31.3)
X^2^ (*p*-value)	3.902 (*p* = 0.142)		0.436 (*p* = 0.804)		4.59 (*p* = 0.101)		2.807 (*p* = 0.246)		0.816 (*p* = 0.665)	
Energy drinks (portion)										
No intake	219 (35.7)	395 (64.3)	338 (55.0)	276 (45.0)	468 (76.2)	146 (23.8)	389 (63.4)	225 (36.6)	432 (70.4)	182 (29.6)
1–4/week	45 (39.8)	68 (60.2)	65 (57.5)	48 (42.5)	96 (85.0)	17 (15.0)	74 (65.5)	39 (34.5)	86 (76.1)	27 (23.9)
5 or more/week	23 (45.1)	28 (54.9)	34 (66.7)	17 (33.3)	43 (84.3)	8 (15.7)	38 (74.5)	13 (25.5)	42 (82.4)	9 (17.6)
X^2^ (*p*-value)	2.287 (*p* = 0.319)		2.68 (*p* = 0.262)		5.506 (*p* = 0.064)		2.624 (*p* = 0.269)		4.475 (*p* = 0.107)	
Hibiscus drinks (portion)										
No intake	271 (38.4)	435 (61.6)	406 (57.5)	300 (42.5)	557 (78.9)	149 (21.1)	460 (65.2)	246 (34.8)	516 (73.1)	190 (26.9)
1–4/week	12 (20.7)	46 (79.3)	24 (41.4)	34 (58.6)	40 (69.0)	18 (31.0)	34 (58.6)	24 (41.4)	36 (62.1)	22 (37.9)
5 or more/week	4 (28.6)	10 (71.4)	7 (50.0)	7 (50.0)	10 (71.4)	4 (28.6)	7 (50.0)	7 (50.0)	8 (57.1)	6 (42.9)
X^2^ (*p*-value)	**7.633** **(*p* = 0.022)**		5.883 (*p* = 0.053)		3.443 (*p* = 0.179)		2.287 (*p* = 0.319)		4.782 (*p* = 0.092)	
Cinnamon drink (portion)										
No intake	268 (38.6)	426 (61.4)	398 (57.3)	296 (42.7)	549 (79.1)	145 (20.9)	457 (65.9)	237 (34.1)	511 (73.6)	183 (26.4)
1–4/week	11 (21.2)	41 (78.8)	22 (42.3)	30 (57.7)	36 (69.2)	16 (30.8)	24 (46.2)	28 (53.8)	29 (55.8)	23 (44.2)
5 or more/week	8 (25.0)	24 (75.0)	17 (53.1)	15 (46.9)	22 (68.8)	10 (31.3)	20 (62.5)	12 (37.5)	20 (62.5)	12 (37.5)
X^2^ (*p*-value)	**8.363 (*p* = 0.015)**		4.571 (*p* = 0.102)		4.424 (*p* = 0.109)		**8.238 (*p* = 0.016)**		**9.139 (*p* = 0.01)**	

Data are shown as frequency and percentages. χ^2^ is the Chi-square test value followed by its *p*-value. Significant differences between groups are shown in bold font LDL-C, low-density lipoprotein cholesterol; HDL-C, high-density lipoprotein cholesterol; TC, total cholesterol; TG, triglycerides.

**Table 3:** Unadjusted and adjusted Odds Ratios (OR) with its 95% Confidence Interval (CI) for dietary and lifestyle predictors of dyslipidemia in men.

Covariate	Unadjusted	Adjusted for Age, BMI and WC
	OR (95% CI) P	OR (95% CI) P
**Turkish coffee intake**		
***p* for trend**	***p* < 0.001**	***p* = 0.001**
No intake (reference)		
1–4 /week	0.955 (0.604, 1.512) *p* = 0.846	0.929 (0.573, 1.507) *p* = 0.766
5 or more /week	**2.783 (1.566, 4.944) *p* < 0.001**	**2.737 (1.532, 4.889) *p* = 0.001**
**American coffee intake**		
***p* for trend**	***p* = 0.005**	***p* = 0.025**
No intake (reference)		
1–4 /week	**0.469 (0.273, 0.805) *p* = 0.006**	0.577 (0.326, 1.021) *p* = 0.059
5 or more /week	**0.544 (0.318, 0.933) *p* = 0.027**	**0.525 (0.298, 0.923) *p* = 0.025**
**Carbonated drinks intake**		
***p* for trend**	*p* > 0.15	***p* = 0.093**
No intake (reference)		
1–4 /week		1.226 (0.815, 1.842) *p* = 0.326
5 or more /week		**1.533 (1.041, 2.257) *p* = 0.03**
**Sleep duration**		
***p* for trend**	***p* = 0.011**	***p* = 0.012**
6–8 h (reference)		
<6 h	**1.566 (1.155, 2.123) *p* = 0.004**	**1.573 (1.137, 2.175) *p* = 0.006**
>8 h	1.545 (0.795, 3.003) *p* = 0.2	1.844 (0.88, 3.864) *p* = 0.105
**Smoking**		
***p* for trend**	*p* > 0.15	*p* = 0.073
Non-smokers (reference)		
Smokers		**1.41 (1.001, 1.986) *p* = 0.043**
Previous smokers		0.755 (0.381, 1.497) *p* = 0.422

Variables having a *p* > 0.15 in the initial logistic regression analysis between the dependent and independent variables were not included in the stepwise regression model. Significant differences between groups are shown in bold font.

**Table 4:** Comparison of dietary habits between normolipidemic and dyslipidemic women groups presented as the number of women and (% of the group) for overall dyslipidemia, and abnormalities in different lipid fractions.

Variable	General Dyslipidemia		LDL-C		HDL-C		TC		TG	
	No (*n* = 240)	Yes (*n* = 367)	Normal (*n* = 381)	High (*n* = 226)	Normal (*n* = 440)	Low (*n* = 167)	Normal (*n* = 404)	High (*n* = 203)	Normal (*n* = 524)	High (*n* = 82)
	N (%)	N (%)	N (%)	N (%)	N (%)	N (%)	N (%)	N (%)	N (%)	N (%)
Fruit (portion)										
No intake	33 (47.1)	37 (52.9)	47 (67.1)	23 (32.9)	56 (80.0)	14 (20.0)	53 (75.7)	17 (24.3)	61 (87.1)	9 (12.9)
1–4/week	123 (38.2)	199 (61.8)	209 (64.9)	113 (35.1)	219 (68.0)	103 (32.0)	221 (68.6)	101 (31.4)	284 (88.2)	38 (11.8)
5 or more/week	84 (39.1)	131 (60.9)	125 (58.1)	90 (41.9)	165 (76.7)	50 (23.3)	130 (60.5)	85 (39.5)	179 (83.3)	36 (16.7)
X^2^ (*p*-value)	1.955 (*p* = 0.372)		3.174 (*p* = 0.204)		**7.168 (*p* = 0.028)**		**6.846 (*p* = 0.033)**		2.713 (*p* = 0.258)	
Fresh vegetable (portion)										
No intake	33 (62.3)	20 (37.7)	42 (79.2)	11 (20.8)	44 (83.0)	9 (17.0)	45 (84.9)	8 (15.1)	50 (94.3)	3 (5.7)
1–4/week	79 (35.3)	145 (64.7)	137 (61.2)	87 (38.8)	152 (67.9)	72 (32.1)	150 (67.0)	74 (33.0)	194 (86.6)	30 (13.4)
5 or more/week	128 (28.8)	202 (61.2)	202 (61.2)	128 (38.8)	244 (73.9)	86 (26.1)	209 (63.3)	121 (36.7)	280 (84.8)	50 (15.2)
X^2^ (*p*-value)	**13.237 (*p* <0.001)**		**6.747 (*p* = 0.034)**		5.704 (*p* = 0.058)		**9.574 (*p* = 0.008)**		3.509 (*p* = 0.173)	
Cooked vegetable (portion)										
No intake	32 (48.5)	34 (51.5)	42 (63.6)	24 (36.4)	53 (80.3)	13 (19.7)	51 (77.3)	15 (22.7)	58 (87.9)	8 (12.1)
1–4/week	100 (36.1)	177 (63.9)	172 (62.1)	105 (37.9)	195 (70.4)	82 (29.6)	176 (63.5)	101 (36.5)	237 (85.6)	40 (14.4)
5 or more/week	108 (40.9)	156 (59.1)	167 (63.3)	97 (36.7)	192 (72.7)	72 (27.3)	177 (67.0)	87 (33.0)	229 (86.7)	35 (13.3)
X^2^ (*p*-value)	3.786 (*p* = 0.151)		0.102 (*p* = 0.95)		2.636 (*p* = 0.268)		4.567 (*p* = 0.102)		0.311 (*p* = 0.856)	
Whole grains (portion)										
No intake	40 (44.0)	51 (56.0)	61 (67.0)	30 (33.0)	64 (70.3)	27 (29.7)	66 (72.5)	25 (27.5)	81 (89.0)	10 (11.0)
1–4/week	34 (37.4)	57 (62.6)	54 (59.3)	37 (40.7)	65 (71.4)	26 (28.6)	57 (62.6)	34 (37.4)	76 (83.5)	15 (16.5)
5 or more/week	166 (39.1)	259 (60.9)	266 (62.6)	159 (37.4)	311 (73.2)	114 (26.8)	281 (66.1)	144 (33.9)	367 (86.4)	58 (13.6)
X^2^ (*p*-value)	0.964 (*p* = 0.618)		1.172 (*p* = 0.557)		0.365 (*p* = 0.833)		2.122 (*p* = 0.346)		1.165 (*p* = 0.559)	
Red meat (portion)										
No intake	65 (46.8)	74 (53.2)	92 (66.2)	47 (33.8)	105 (75.5)	34 (24.5)	95 (68.3)	44 (31.9)	122 (87.8)	17 (12.2)
1–4/week	113 (33.7)	222 (66.3)	204 (60.9)	131 (39.1)	231 (69.0)	104 (31.0)	217 (64.8)	118 (35.2)	287 (85.7)	48 (14.3)
5 or more/week	62 (46.6)	71 (53.4)	85 (63.9)	48 (36.1)	104 (78.2)	29 (21.8)	92 (69.2)	41 (30.8)	115 (86.5)	18 (13.5)
X^2^ (*p*-value)	**10.547 (*p* = 0.005)**		1.272 (*p* = 0.529)		4.918 (*p* = 0.086)		1.086 (*p* = 0.581)		0.369 (*p* = 0.831)	
Fresh juice (portion)										
No intake	78 (42.2)	107 (57.8)	118 (63.8)	67 (36.2)	134 (72.4)	51 (27.6)	127 (68.6)	58 (31.4)	167 (90.3)	18 (9.7)
1–4/week	103 (36.9)	176 (63.1)	174 (62.4)	105 (37.6)	198 (71.0)	81 (29.0)	181 (64.9)	98 (35.1)	236 (84.6)	43 (15.4)
5 or more/week	59 (41.3)	84 (58.7)	89 (62.2)	54 (37.8)	108 (75.5)	35 (24.5)	96 (67.1)	47 (32.9)	121 (84.6)	22 (15.4)
X^2^ (*p*-value)	1.511 (*p* = 0.47)		0.118 (*p* = 0.943)		0.985 (*p* = 0.611)		0.74 (*p* = 0.691)		3.507 (*p* = 0.173)	
Non fresh juice (portion)										
No intake	93 (36.0)	165 (64.0)	158 (61.2)	100 (38.8)	183 (70.9)	75 (29.1)	160 (62.0)	98 (38.0)	220 (85.3)	38 (14.7)
1–4/week	74 (38.9)	116 (61.1)	123 (64.7)	67 (35.3)	136 (71.6)	54 (28.4)	134 (70.5)	56 (29.5)	164 (86.3)	26 (13.7)
5 or more/week	73 (45.9)	86 (54.1)	100 (62.9)	59 (37.1)	121 (76.1)	38 (23.9)	110 (69.2)	49 (30.8)	140 (88.1)	19 (11.9)
X^2^ (*p*-value)	4.046 (*p* = 0.132)		0.574 (*p* = 0.751)		1.433 (*p* = 0.488)		4.228 (*p* = 0.121)		0.644 (*p* = 0.725)	
Carbonated drinks (portion)										
No intake	113 (38.2)	183 (61.8)	182 (61.5)	114 (38.5)	207 (69.9)	89 (30.1)	193 (65.2)	103 (34.8)	248 (83.8)	48 (16.2)
1–4/week	68 (39.1)	106 (60.9)	108 (62.1)	66 (37.9)	130 (74.7)	44 (25.3)	115 (66.1)	59 (33.9)	156 (89.7)	18 (10.3)
5 or more/week	59 (43.1)	78 (56.9)	91 (66.4)	46 (33.6)	103 (75.2)	34 (24.8)	96 (70.1)	41 (29.9)	120 (87.6)	17 (12.4)
X^2^ (*p*-value)	0.958 (*p* = 0.619)		1.028 (*p* = 0.598)		1.9 (*p* = 0.387)		1.022 (*p* = 0.6)		3.44 (*p* = 0.179)	
Red tea (portion)										
No intake	79 (43.4)	103 (56.6)	125 (68.7)	57 (31.3)	129 (70.9)	53 (29.1)	136 (74.7)	46 (25.3)	161 (88.5)	21 (11.5)
1–4/week	42 (32.1)	89 (67.9)	75 (57.3)	56 (42.7)	91 (69.5)	40 (30.5)	77 (58.8)	54 (41.2)	110 (84.0)	21 (16.0)
5 or more/week	119 (40.5)	175 (59.5)	181 (61.6)	113 (38.4)	220 (74.8)	74 (25.2)	191 (65.0)	103 (35.0)	253 (86.1)	41 (13.9)
X^2^ (*p*-value)	4.311 (*p* = 0.116)		4.611 (*p* = 0.1)		1.645 (*p* = 0.439)		**9.351 (*p* = 0.009)**		1.338 (*p* = 0.512)	
Green tea (portion)										
No intake	134 (41.9)	186 (58.1)	215 (67.2)	105 (32.8)	227 (70.9)	93 (29.1)	226 (70.6)	94 (29.4)	281 (87.8)	39 (12.2)
1–4/week	50 (38.5)	80 (61.5)	75 (57.7)	55 (42.3)	94 (72.3)	36 (27.7)	85 (65.4)	45 (34.6)	108 (83.1)	22 (16.9)
5 or more/week	56 (35.7)	101 (64.3)	91 (58.0)	66 (42.0)	119 (75.8)	38 (24.2)	93 (59.2)	64 (40.8)	135 (86.0)	22 (14.0)
X^2^ (*p*-value)	1.777 (*p* = 0.411)		5.659 (*p* = 0.059)		1.249 (*p* = 0.535)		**6.24 (*p* = 0.044)**		1.777 (*p* = 0.411)	
Arabic coffee (portion)										
No intake	93 (40.4)	137 (59.6)	148 (64.3)	82 (35.7)	161 (70.0)	69 (30.0)	159 (69.1)	71 (30.9)	199 (86.5)	31 (13.5)
1–4/week	57 (37.3)	96 (62.7)	98 (64.1)	55 (35.9)	104 (68.0)	49 (32.0)	104 (68.0)	49 (32.0)	130 (85.0)	23 (15.0)
5 or more/week	90 (40.2)	134 (59.8)	135 (60.3)	89 (39.7)	175 (78.1)	49 (21.9)	141 (62.9)	83 (37.1)	195 (87.1)	29 (12.9)
X^2^ (*p*-value)	0.449 (*p* = 0.799)		0.953 (*p* = 0.621)		5.846 (*p* = 0.054)		2.134 (*p* = 0.344)		0.347 (*p* = 0.841)	
Turkish coffee (portion)										
No intake	170 (39.1)	265 (60.9)	280 (64.4)	155 (35.6)	310 (71.3)	125 (28.7)	284 (65.3)	151 (34.7)	378 (86.9)	57 (13.1)
1–4/week	31 (33.7)	61 (66.3)	52 (56.5)	40 (43.5)	65 (70.7)	27 (29.3)	64 (69.6)	28 (30.4)	77 (83.7)	15 (16.3)
5 or more/week	39 (48.8)	41 (51.2)	49 (61.3)	31 (38.8)	65 (81.3)	15 (18.8)	56 (70.0)	24 (30.0)	69 (86.3)	11 (13.8)
X^2^ (*p*-value)	4.192 (*p* = 0.123)		2.091 (*p* = 0.351)		3.562 (*p* = 0.168)		1.115 (*p* = 0.573)		0.66 (*p* = 0.719)	
American coffee (portion)										
No intake	212 (40.2)	315 (59.8)	332 (63.0)	195 (37.0)	382 (72.5)	145 (27.5)	353 (67.0)	174 (33.0)	460 (87.3)	67 (12.7)
1–4/week	12 (27.9)	31 (72.1)	27 (62.8)	16 (37.2)	27 (62.8)	16 (37.2)	30 (69.8)	13 (30.2)	34 (79.1)	9 (20.9)
5 or more/week	16 (43.2)	21 (56.8)	22 (59.5)	15 (40.5)	31 (83.8)	6 (16.2)	21 (56.8)	16 (43.2)	30 (81.1)	7 (18.9)
X^2^ (*p*-value)	2.751 (*p* = 0.253)		0.185 (*p* = 0.912)		4.395 (*p* = 0.111)		1.839 (*p* = 0.300)		3.192 (*p* = 0.203)	
Cappuccino (portion)										
No intake	140 (38.6)	223 (61.4)	216 (59.5)	147 (40.5)	255 (70.2)	108 (29.8)	238 (65.6)	125 (34.4)	306 (84.3)	57 (15.7)
1–4/week	60 (43.2)	79 (56.8)	99 (71.2)	40 (28.8)	104 (74.8)	35 (25.2)	98 (70.5)	41 (29.5)	123 (88.5)	16 (11.5)
5 or more/week	40(38.1)	65 (61.9)	66 (62.9)	39 (37.1)	81 (77.1)	24 (22.9)	68 (64.8)	37 (35.2)	95 (90.5)	10 (9.5)
X^2^ (*p*-value)	1 (*p* = 0.607)		5.907 (*p* = 0.052)		2.433 (*p* = 0.296)		1.285 (*p* = 0.526)		3.349 (*p* = 0.187)	
Energy drinks (portion)										
No intake	212 (39.3)	328 (60.7)	334 (61.9)	206 (38.1)	388 (71.9)	152 (28.1)	356 (65.9)	184 (34.1)	464 (85.9)	76 (24.1)
1–4/week	15 (33.3)	30 (66.7)	31 (68.9)	14 (31.1)	32 (71.1)	13 (28.9)	30 (66.7)	15 (33.3)	40 (88.9)	5 (11.1)
5 or more/week	13 (59.1)	9 (40.9)	16 (72.7)	6 (27.3)	20 (90.9)	2 (9.1)	18 (81.8)	4 (18.2)	20 (90.0)	2 (9.1)
X^2^ (*p*-value)	2.261 (*p* = 0.119)		1.849 (*p* = 0.397)		3.896 (*p* = 0.143)		2.399 (*p* = 0.301)		0.715 (*p* = 0.699)	
Hibiscus drinks (portion)										
No intake	220 (39.9)	331 (60.1)	352 (63.9)	199 (36.1)	396 (71.9)	155 (28.1)	374 (67.9)	177 (32.1)	474 (86.0)	77 (14.0)
1–4/week	15 (35.7)	27 (64.3)	21 (50.0)	21 (50.0)	32 (76.2)	10 (23.8)	24 (57.1)	18 (42.9)	38 (90.5)	4 (9.5)
5 or more/week	5 (35.7)	9 (64.3)	8 (57.1)	6 (42.9)	12 (85.7)	2 (14.3)	6 (42.9)	8 (57.1)	12 (85.7)	2 (14.3)
X^2^ (*p*-value)	0.377 (*p* = 0.828)		3.413 (*p* = 0.182)		1.622 (*p* = 0.444)		5.636 (*p* = 0.06)		0.659 (*p* = 0.719)	
Cinnamon drink (portion)										
No intake	200 (42.2)	274 (57.8)	310 (65.4)	164 (34.6)	348 (73.4)	126 (26.6)	328 (69.2)	146 (30.8)	412 (86.9)	62 (13.1)
1–4/week	32 (32.30)	67 (67.7)	54 (54.5)	45 (45.5)	70 (70.7)	29 (29.3)	62 (62.6)	37 (37.4)	85 (85.9)	14 (14.1)
5 or more/week	8 (23.5)	26 (76.5)	17 (50.0)	17 (50.0)	22 (64.7)	12 (35.3)	14 (41.2)	20 (58.8)	27 (79.4)	7 (20.6)
X^2^ (*p*-value)	**7.199 (*p* = 0.027)**		**6.642 (*p* = 0.036)**		1.395 (*p* = 0.498)		**12.013 (*p* = 0.002)**		1.537 (*p* = 0.464)	

Data are shown as frequency and percentages. χ^2^ is the Chi-square test value followed by its *p*-value. Significant differences between groups are shown in bold font. LDL-C, low-density lipoprotein cholesterol; HDL-C, high-density lipoprotein cholesterol; TC, total cholesterol; TG, triglycerides.

**Table 5:** Unadjusted and adjusted Odds Ratio (OR) with its 95% Confidence Interval (CI) for the predictors of dyslipidemia in women.

Covariate	Unadjusted	Adjusted for Age, BMI and WC
	OR (95% CI) P	OR (95% CI) P
**Fresh vegetables intake**		
***p* for trend**	***p* = 0.007**	***p* = 0.024**
No intake (reference)		
1–4/week	**2.683 (1.416, 5.085) *p* = 0.002**	**2.499 (1.285, 4.859) *p* = 0.007**
5 or more/week	**2.489 (1.34, 4.624) *p* = 0.004**	**2.072 (1.089, 3.941) *p* = 0.026**
**Cinnamon drink intake**		
***p* for trend**	***p* = 0.027**	*p* > 0.15
No intake (reference)		
1–4/week	1.395 (0.873, 2.228) *p* = 0.163	
5 or more/week	**2.613 (1.142, 5.978) *p* = 0.023**	

Variables having a *p* > 0.15 in the initial logistic regression analysis between the dependent and independent variables were not included in the stepwise regression model. Significant differences between groups are shown in bold font.

## Supplementary Materials

The following are available online at https://www.mdpi.com/2072-6643/12/8/2441/s1, Figure S1: Recruitment flow diagram, Table S1: Demographic, anthropometric, clinical and biochemical characteristics of studied groups, Table S2: Comparison of demographic, anthropometric, and lifestyle characteristics of normolipidemia vs. dyslipidemia groups presented as number of people (%) for overall dyslipidemia, and abnormalities in different lipid parameters in men (a) and women (b), Table S3: Anthropometric and clinical characteristics of vegetable intake groups in women.

## Abbreviations

BMI, body mass index; BP, blood pressure; CVD, cardiovascular disease; HDL-C, high density lipoprotein cholesterol; LDL-C, low density lipoprotein cholesterol; PHCC, primary health care centers; SD, standard deviation; TC, total cholesterol; TG, triglycerides; WC, waist circumference; WHO, World Health Organization.
