# Correction: Enani et al. The Association Between Dyslipidemia, Dietary Habits and Other Lifestyle Indicators Among Non-Diabetic Attendees of Primary Health Care Centers in Jeddah, Saudi Arabia. *Nutrients* 2020, *12*, 2441

**DOI:** 10.3390/nu18172772

**Published:** 2026-08-25

**Authors:** Sumia Enani, Suhad Bahijri, Manal Malibary, Hanan Jambi, Basmah Eldakhakhny, Jawaher Al-Ahmadi, Rajaa Al Raddadi, Ghada Ajabnoor, Anwar Boraie, Jaakko Tuomilehto

**Affiliations:** 1 Saudi Diabetes Research Group, King Fahd Medical Research Center, King Abdulaziz University, Jeddah 3270, Saudi Arabia; sb@kau.edu.sa (S.B.); mamalibary@kau.edu.sa (M.M.); hjambi@kau.edu.sa (H.J.); beldakhakhny@kau.edu.sa (B.E.); j_al_ahmadi@yahoo.com (J.A.-A.); rmsalharbi@kau.edu.sa (R.A.R.); gajabnour@kau.edu.sa (G.A.); boraiaa@ngha.med.sa (A.B.); jotuomilehto@gmail.com (J.T.); 2 Department of Food and Nutrition, Faculty of Human Sciences and Design, King Abdulaziz University, Jeddah 3270, Saudi Arabia; 3 Department of Clinical Biochemistry, Faculty of Medicine, King Abdulaziz University, Jeddah 22252, Saudi Arabia; 4 Department of Family Medicine, Faculty of Medicine, King Abdulaziz University, Jeddah 22252, Saudi Arabia; 5 Department of Community Medicine, Faculty of Medicine, King Abdulaziz University, Jeddah 22252, Saudi Arabia; 6 King Abdullah International Medical Research Center (KAIMRC), College of Medicine, King Saud Bin Abdulaziz, University for Health Sciences (KSAU-HS), Jeddah 22384, Saudi Arabia; 7 Department of Public Health, University of Helsinki, FI-00014 Helsinki, Finland; 8 Public Health Promotion Unit, Finnish Institute for Health and Welfare, FI-00271 Helsinki, Finland

The authors wish to make the following corrections to the published article [1].

## 1. Correction to Ethics Approval Number

In Sections 2 and 2.1, the ethics approval number was incorrectly reported as: “Reference No. 338-10”. This was stated in error. The correct ethics approval number for the study is: “Reference No. 1270-13”. The correct sentence appears below:

“The Committee on the Ethics of Human Research at the Faculty of Medicine-King Abdulaziz University, Jeddah approved the study (Reference No. 1270-13).”

The study was conducted under this approval, and written informed consent was obtained from all participants as originally described. The previously listed number was included inadvertently during manuscript preparation.

## 2. Stepwise Model Selection and Multiplicity

To clarify the analytical approach and the interpretive status of the findings, the following clarifications are added.

In the Statistical Analysis Section 2.4, the text regarding the *p* < 0.15 threshold is amended to:

“Only related independent variables, where *p* < 0.15 after an initial logistic regression between the dependent and independent variable, were used in the corresponding stepwise regression model. This liberal screening threshold (rather than *p* < 0.05) was used deliberately to avoid premature exclusion of potentially important covariates and to retain predictive variables with a potential or high effect, in line with established model-building guidelines [29]. This lower threshold is limited to variable inclusion only and is not a test of statistical significance.”

A statement on multiplicity is added in Section 4, the second-to-last paragraph; the following limitations are added:

“Given the large number of dietary, lifestyle, and lipid variables examined, numerous statistical comparisons were performed. No formal adjustments were made for these multiple comparisons. Therefore, the associations reported here should be considered exploratory and hypothesis-generating, not confirmatory, and some results may represent random associations. These findings require confirmation in adequately powered, prospectively designed studies.”

## 3. Several Clarifications and Corrections Have Been Made in Section 3

First, the denominator for Table 1 and the biochemical analyses in the second sentence were not stated clearly. The Results section has been updated to clarify that, of 1477 recruited participants, 1403 completed the dietary questionnaire, which is the denominator for Table 1, and biochemical lipid profile data were available for 1385 participants, who were used for the dyslipidemia analyses. The corrected sentence appears below:

“Complete dietary questionnaire data were obtained from 1403 people, while biochemical lipid profile data were available for 1385 people.”

Second, the wording regarding missing data in the first paragraph of the Results section has been corrected. The original text stated that the missing data “was completely random and is not expected to affect validity.” This wording is corrected to read:

“Missing data were mainly due to missing, hemolysed, broken, or unlabeled blood samples, and there is no indication that this is related to participant characteristics or lipid status. Therefore, they are presumed to be missing at random; although this could not be directly verified, the proportion of missing data was small and unlikely to materially affect the validity of the results.”

Third, the description of the most prevalent type of dyslipidemia contained a typographical error, in which the count for women was incorrectly labeled as men. The original sentence stated that elevated LDL-C levels were found in 567 individuals (40.9%) (341 men and 226 men). The correct version is:

“The most prevalent type of dyslipidemia was high LDL-C found in 567 people (40.9%) (341 men and 226 women), followed by high TC in 480 people (34.7%) (277 men and 203 women), and low HDL-C in 338 people (24.4%) (171 men and 167 women) and the least prevalent type of dyslipidemia was high TG levels in 301 people (21.7%) (218 men and 83 women).”

The footnote of Table 1 has also been updated to state these denominators explicitly and appears below:

“Data is shown as frequencies and percentages of all people in the sex group. χ^2^ is the chi-square test value followed by its *p*-value. Significant differences between groups are shown in bold font. This table is based on participants with completed dietary questionnaire data (*n* = 1403). Analyses involving lipid outcomes are restricted to participants with available biochemical lipid profile data (*n* = 1385).”

The authors state that the scientific conclusions are unaffected. This correction was approved by the Academic Editor. The original publication has also been updated.
