# Supplementary material for: The Association between Dyslipidemia, Dietary Habits and Other Lifestyle Indicators among Non-Diabetic Attendees of Primary Health Care Centers in Jeddah, Saudi Arabia
Source: Nutrients. 2020 Aug 13;12(8):2441. doi: 10.3390/nu12082441 (PMC7469008; doi:10.3390/nu12082441)
Supplement: Supplementary file 1 [file nutrients-12-02441-s001.pdf]

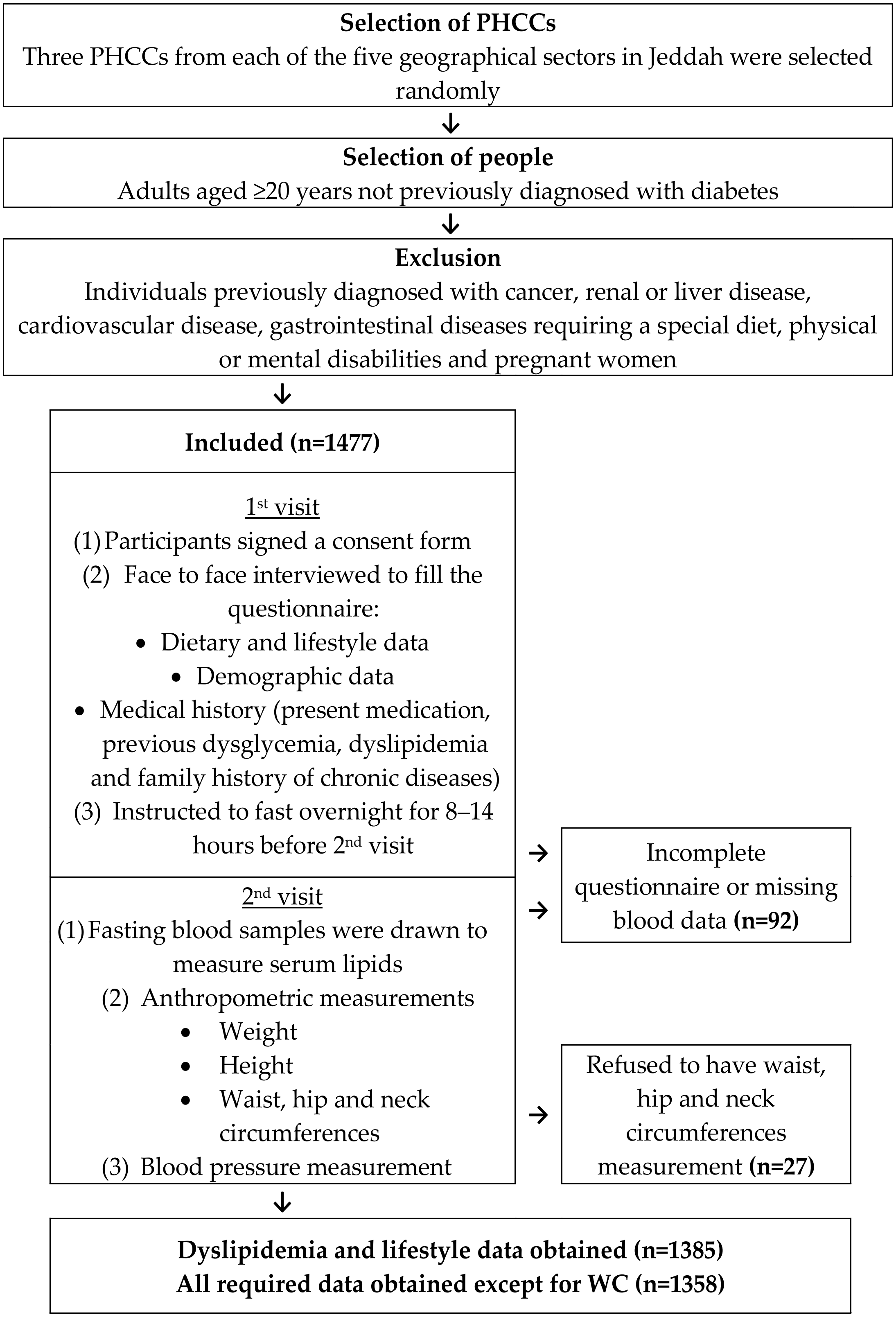

Figure 1. Recruitment flow diagram.

Table S1. Demographic, anthropometric, clinical and biochemical characteristics of studied groups

	Men		Women	
	**Normolipidemia** n = 287	**Dyslipidemia** n = 491	**Normolipidemia** n = 240	**Dyslipidemia** n = 367
	Mean ± SD	Mean ± SD	Mean ± SD	Mean ± SD
**Anthropometric measurements**				
Age-yrs	28 ± 8.4	34 ± 11.3***	30 ± 11.3	36 ± 12.3***
BMI	26.0 ± 5.7	28.7 ± 6.0***	25.6 ± 6.2	29 ± 6.3 ***
Weight (kg)	76.5 ± 16.9	84.4 ± 19.1***	64.1 ± 16.2	71.8 ± 15.9***
Fat (%)	24.3 ± 9.7	28.9 ± 8.9***	36.3 ± 11.3	42.2 ± 10.7***
Neck circ. (cm)	38.8 ± 4.1	39.9 ± 4.3***	32.4 ± 3.7	34.4 ± 4.2***
Waist circ. (cm)	92.3 ± 14.5	99.8 ± 15.6***	82.7 ± 14.8	92.3 ± 15.7***
Hip circ. (cm)	104.3 ± 12.5	108.9 ± 14.2***	101.6 ± 12.3	108.0 ± 14.0***
Waist to hip ratio	0.88 ± 0.08	0.92 ± 0.08***	0.81 ± 0.09	0.85 ± 0.09***
Waist to height ratio	0.54 ± 0.09	0.58 ± 0.09***	0.52 ± 0.1	0.59 ± 0.1***
**Clinical measurements**				
BP-Systolic	119 ± 11.7	123 ± 14.7***	110 ± 13.6	115 ± 16.4***
BP-Diastolic	73 ± 11.7	77 ± 11.0***	69 ± 11.86	72 ± 11.3***
**Serum biochemical measurements**				
TC (mmol/l)	4.2 ± 0.5	5.18 ± 0.9***	4.8 ± 0.5	5.2 ± 1.1***
HDL-C (mmol/l)	1.4 ± 0.2	1.19 ± 0.3***	1.6 ± 0.2	1.4 ± 0.3***
TG (mmol/l)	0.8 ± 0.3	1.81 ± 1.2***	0.8 ± 0.3	1.3 ± 0.7***
LDL-C (mmol/l)	2.6 ± 0.5	3.64 ± 0.9***	2.6 ± 0.5	3.5 ± 0.9***

Data are presented as mean ± SD. Differences in measurements between individuals with and without dyslipidemia was analyzed using student’s t-test.

*** denotes significant association with dyslipidemia (P ≤0.001)

BP, blood pressure; TC, total cholesterol; HDL-C, high density lipoprotein cholesterol; LDL-C, low density lipoprotein cholesterol; TG, triglycerides

Fat percentage was measured using a portable calibrated scale.

Table S2. Comparison of demographic, anthropometric, and lifestyle characteristics of normolipidemia vs. dyslipidemia groups presented as number of people (%) for overall dyslipidemia, and abnormalities in different lipid parameters in men (a) and women (b)

(a)

Variable	General Dyslipidemia		LDL-C		HDL-C		TC		TG	
	Normal (n=287)	Dyslipidemia (n=491)	Normal (n=437)	High (n=341)	Normal (n=607)	Low (n=171)	Normal (n=501)	High (n=227)	Normal (n=560)	High (n=218)
	N (%)	N (%)	N (%)	N (%)	N (%)	N (%)	N (%)	N (%)	N (%)	N (%)
**Age**										
<30	203 (49.4)	208 (50.6)	265 (64.5)	146 (35.5)	352 (85.6)	59 (14.4)	306 (74.5)	105 (25.5)	347 (84.4)	64 (15.6)
30+	84 (22.9)	283 (77.1)	172 (46.9)	195 (53.1)	255 (69.5)	112 (30.5)	195 (53.1)	172 (46.9)	213 (58)	154 (42)
X^2 (P-value)	**58.496 (P <0.001)**		**24.423 (P <0.001)**		**29.534 (P <0.001)**		**38.433 (P <0.001)**		**66.946 (P <0.001)**	
**BMI**										
Underweight	17 (70.8)	7 (29.2)	21 (87.5)	3 (12.5)	21 (87.5)	3 (12.5)	21 (87.5)	3 (12.5)	22 (91.7)	2 (8.3)
Normal	112 (46.5)	129 (53.5)	147 (61)	94 (39)	201 (83.4)	40 (16.6)	164 (68)	77 (32)	195 (80.9)	46 (19.1)
Overweight	101 (35.2)	186 (64.8)	159 (55.4)	128 (44.6)	223 (77.7)	64 (22.3)	184 (64.1)	103 (35.9)	200 (69.7)	87 (30.3)
Obese	57 (25.2)	169 (74.8)	110 (48.7)	116 (51.3)	162 (71.7)	64 (28.3)	132 (58.4)	94 (41.6)	143 (63.3)	83 (36.7)
X^2 (P-value)	**34.957 (P <0.001)**		**17.078 (P =0.001)**		**10.641 (P =0.014)**		**10.537 (P =0.015)**		**23.388 (P <0.001)**	
**WC**										
Normal	167 (47.2)	187 (52.8)	224 (63.3)	130 (36.7)	295 (83.3)	59 (16.7)	251 (70.9)	103 (29.1)	284 (80.2)	70 (19.8)
Level 1	67 (37)	114 (63)	100 (55.2)	81 (44.8)	146 (80.7)	35 (19.3)	110 (60.8)	71 (39.2)	132 (72.9)	49 (27.1)
Level 2	50 (21.4)	184 (78.6)	109 (46.6)	125 (53.4)	160 (68.4)	74 (31.6)	134 (57.3)	100 (42.7)	138 (59)	96 (41)
X^2 (P-value)	**40.285 (P <0.001)**		**16.069 (P <0.001)**		**19.332 (P <0.001)**		**12.761 (P =0.002)**		**31.681 (P <0.001)**	
**Physical activity of at least 30 minutes per day for at least 5 days per week**										
No	147 (34.4)	280 (65.6)	233 (54.6)	194 (45.4)	324 (75.9)	103 (24.1)	260 (60.9)	167 (39.1)	295 (69.1)	132 (30.9)
Yes	140 (39.9)	211 (60.1)	204 (58.1)	147 (41.9)	283 (80.6)	68 (19.4)	241 (68.7)	110 (31.3)	265 (75.5)	86 (24.5)
X^2 (P-value)	2.467 (P =0.116)		0.988 (P =0.32)		2.533 (P =0.111)		**5.074 (P =0.024)**		**3.927 (P =0.048)**	

**Sleeping duration**										
<6 hours	105 (31.5)	228 (68.5)	174 (52.3)	159 (47.7)	254 (76.3)	79 (23.7)	197 (59.2)	136 (40.8)	237 (71.2)	96 (28.8)
6-8 hours	168 (41.9)	233 (58.1)	241 (60.1)	160 (39.9)	320 (79.8)	81 (20.2)	273 (68.1)	128 (31.9)	289 (72.1)	112 (27.9)
>8 hours	14 (31.8)	30 (68.2)	22 (50)	22 (50)	33 (75)	11 (25)	31 (70.5)	13 (29.5)	34 (77.3)	10 (22.7)
X^2 (P-value)	**8.908 (P =0.012)**		5.272 (P =0.072)		1.566 (P =0.457)		**7.061 (P =0.029)**		0.721 (P =0.697)	
**Sitting hours/day**										
Less than 4	41 (39.4)	63 (60.6)	61 (58.7)	43 (41.3)	74 (71.2)	30 (28.8)	68 (65.4)	36 (34.6)	78 (75)	26 (25)
4 – 5	93 (39.4)	143 (60.6)	145 (61.4)	91 (38.6)	191 (80.9)	45 (19.1)	161 (68.2)	75 (31.8)	160 (67.8)	76 (32.2)
6 – 8	96 (34.8)	180 (65.2)	146 (52.9)	130 (47.1)	216 (78.3)	60 (21.7)	176 (63.8)	100 (36.2)	209 (75.7)	67 (24.3)
more than 8	57 (35.2)	105 (64.8)	85 (52.5)	77 (47.5)	126 (77.8)	36 (22.2)	96 (59.3)	66 (40.7)	113 (69.8)	49 (30.2)
X^2 (P-value)	1.657 (P =0.646)		5.025 (P =0.17)		4.041 (P =0.257)		3.462 (P =0.326)		4.835 (P =0.184)	
**Smoking habits**										
Non-smoker	186 (38.9)	292 (61.1)	279 (58.4)	199 (41.6)	378 (79.1)	100 (20.9)	314 (65.7)	164 (34.3)	355 (74.3)	123 (25.7)
Smoker	83 (32.3)	174 (67.7)	134 (52.1)	123 (47.9)	195 (75.9)	62 (24.1)	161 (62.6)	96 (37.4)	169 (65.8)	88 (34.2)
Previous smoker	18 (41.9)	25 (58.1)	24 (55.8)	19 (44.2)	34 (79.1)	9 (20.9)	26 (60.5)	17 (39.5)	36 (83.7)	7 (16.3)
X^2 (P-value)	3.626 (P =0.163)		2.636 (P =0.268)		1.03 (P =0.598)		0.982 (P =0.612)		**9.111 (P =0.011)**	

(b)

Variable	**General Dyslipidemia**		**LDL-C**		**HDL-C**		**TC**		**TG**	
	Normal (n=240)	Dyslipidemia (n=367)	Normal (n=437)	High (n=381)	Normal (n=226)	Low (n=167)	Normal (n=404)	High (n=203)	Normal (n=524)	High (n=83)
	N (%)	N (%)	N (%)	N (%)	N (%)	N (%)	N (%)	N (%)	N (%)	N (%)
**Age**										
<30	151 (53.7)	130 (46.3)	207 (73.7)	74 (26.3)	223 (79.4)	58 (20.6)	222 (79)	59 (21)	266 (94.7)	15 (5.3)
30+	89 (27.3)	237 (72.7)	174 (53.4)	152 (46.6)	217 (66.6)	109 (33.4)	182 (55.8)	144 (44.2)	258 (79.1)	68 (20.9)
X^2 (P-value)	**44.119 (P <0.001)**		**26.589 (P <0.001)**		**12.389 (P <0.001)**		**36.416 (P <0.001)**		**30.799 (P <0.001)**	
**BMI**										

Underweight	17 (68)	8 (32)	19 (76)	6 (24)	23 (92)	2 (8)	23 (92)	2 (8)	24 (96)	1 (4)
Normal	112 (56.3)	87 (43.7)	158 (79.4)	41 (20.6)	162 (81.4)	37 (18.6)	154 (77.4)	45 (22.6)	190 (95.5)	9 (4.5)
Overweight	59 (29.9)	138 (70.1)	114 (57.9)	83 (42.1)	134 (68)	63 (32)	126 (64)	71 (36)	165 (83.8)	32 (16.2)
Obese	52 (28)	134 (72)	90 (48.4)	96 (51.6)	121 (65.1)	65 (34.9)	101 (54.3)	85 (45.7)	145 (78)	41 (22)
X^2 (P-value)	**49.821 (P <0.001)**		**43.904 (P <0.001)**		**19.837 (P <0.001)**		**30.906 (P <0.001)**		**28.239 (P <0.001)**	
**WC**										
Normal	118 (56.2)	92 (43.8)	163 (77.6)	47 (22.4)	171 (81.4)	39 (18.6)	167 (79.5)	43 (20.5)	202 (96.2)	8 (3.8)
Level 1	40 (42.1)	55 (57.9)	65 (68.4)	30 (31.6)	67 (70.5)	28 (29.5)	70 (73.7)	25 (26.3)	87 (91.6)	8 (8.4)
Level 2	72 (25.4)	212 (74.6)	141 (49.6)	143 (50.4)	188 (66.2)	96 (33.8)	156 (54.9)	128 (45.1)	221 (77.8)	63 (22.2)
X^2 (P-value)	**48.684 (P <0.001)**		**41.979 (P <0.001)**		**14.177 (P =0.001)**		**35.362 (P <0.001)**		**37.524 (P <0.001)**	
**Physical activity of at least 30 minutes per day for at least 5 days per week**										
No	130 (37)	221 (63)	207 (59)	144 (41)	255 (72.6)	96 (27.4)	226 (64.4)	125 (35.6)	299 (85.2)	52 (14.8)
Yes	110 (43)	146 (57)	174 (68)	82 (32)	185 (72.3)	71 (27.7)	178 (69.5)	78 (30.5)	225 (87.9)	31 (12.1)
X^2 (P-value)	2.179 (P =0.14)		**5.124 (P =0.024)**		0.011 (P =0.917)		1.76 (P =0.185)		0.918 (P =0.338)	
**Sleeping duration**										
<6 hours	78 (36.6)	135 (63.4)	123 (57.7)	90 (42.3)	155 (72.8)	58 (27.2)	131 (61.5)	82 (38.5)	178 (83.6)	35 (16.4)
6-8 hours	129 (41.9)	179 (58.1)	206 (66.9)	102 (33.1)	221 (71.8)	87 (28.2)	215 (69.8)	93 (30.2)	270 (87.7)	38 (12.3)
>8 hours	33 (38.4)	53 (61.6)	52 (60.5)	34 (39.5)	64 (74.4)	22 (25.6)	58 (67.4)	28 (32.6)	76 (88.4)	10 (11.6)
X^2 (P-value)	1.516 (P =0.469)		4.725 (P =0.094)		0.253 (P =0.881)		3.935 (P =0.14)		2.143 (P =0.342)	
**Sitting hours/day**										
Less than 4	52 (33.1)	105 (66.9)	95 (60.5)	62 (39.5)	100 (63.7)	57 (36.3)	96 (61.1)	61 (38.9)	134 (85.4)	23 (14.6)
4 - 5	74 (39.6)	113 (60.4)	116 (62)	71 (38)	139 (74.3)	48 (25.7)	121 (64.7)	66 (35.3)	168 (89.8)	19 (10.2)
6 - 8	70 (44.9)	86 (55.1)	107 (68.6)	49 (31.4)	117 (75)	39 (25)	116 (74.4)	40 (25.6)	130 (83.3)	26 (16.7)
more than 8	44 (41.1)	63 (58.9)	63 (58.9)	44 (41.1)	84 (78.5)	23 (21.5)	71 (66.4)	36 (33.6)	92 (86)	15 (14)
X^2 (P-value)	4.673 (P =0.197)		3.341 (P =0.342)		**8.842 (P =0.031)**		6.621 (P =0.085)		3.277 (P =0.351)	
**Smoking habits**										
Non-smoker	227 (40.2)	338 (59.8)	361 (63.9)	204 (36.1)	410 (72.6)	155 (27.4)	381 (67.4)	184 (32.6)	491 (86.9)	74 (13.1)

Smoker	13 (32.5)	27 (67.5)	20 (50)	20 (50)	28 (70)	12 (30)	23 (57.5)	17 (42.5)	31 (77.5)	9 (22.5)
Previous smoker	0 (P =0)	2 (100)	0 (P =0)	2 (100)	2 (100)	0 (P =0)	0 (P =0)	2 (100)	2 (100)	0 (P =0)
X^2 (P-value)	2.233 (P =0.420)		**6.468 (P =0.035)**		0.885 (P =0.851)		5.649 (P =0.057)		3.116 (P =0.204)	

People were classified according to their BMI as underweight (<18.5 kg/M2), normal 18.5->25 kg/M2, overweight 25-<30 kg/M2 and obese ≥30 kg/M2. People were classified according to their WC as normal adiposity (≤94cm for men, ≤80 cm for women), level 1 (>94cm for men, >80 cm for women) and level 2 (>102cm for men, >88 cm for women). Association between measures and dyslipidemia was analyzed using Chi-square test.

χ2 is the Chi-square test or Fisher's Exact test value followed by its P-value. Significant differences between groups are shown in bold font

BMI, body mass index; WC, waist circumference; LDL-C, low density lipoprotein cholesterol; HDL-C, high density lipoprotein cholesterol; TC, total cholesterol; TG, triglycerides

Table S3. Anthropometric and clinical characteristics of vegetable intake groups in women

	Intake of fresh vegetables		
	**No intake** n = 53	**1-4 portions/week** n = 224	**5 or more portions/week** n = 330
	Mean ± SD	Mean ± SD	Mean ± SD
**Anthropometric measurements** [a]			
Age-yrs	27 ± 10.9	34 ± 12.1**	35 ± 12.4**
BMI	26.4 ± 7.1	27.86 ± 6.6	27.6 ± 6.3
Weight (kg)	63.2 ± 16.1	69.81 ± 17.3*	68.8 ± 15.8*
Fat (%)	37.4 ± 10.6	40 ± 11.9	40.2 ± 10.9
Neck circ. (cm)	32.3 ± 4.3	33.67 ± 4.3*	33.9 ± 4.1*
Waist circ. (cm)	85.2 ± 15.9	89.67 ± 16.3	88.2 ± 15.8
Hip circ. (cm)	102.6 ± 15.4	106.22 ± 14.1	105.3 ± 13.2
Waist to hip ratio	0.83 ± 0.1	0.84 ± 0.09	0.84 ± 0.09
Waist to height ratio	0.55 ± 0.1	0.57 ± 0.10	0.56 ± 0.10
**Clinical measurements** [a]			
BP-Systolic	116 ± 18.1	114 ± 13.4	112 ± 16.35
BP-Diastolic	71 ± 11.3	72 ± 12.3	70 ± 11.27
**Previous diagnosis of high TC** [b]			
No	51 (96.2)	198 (88.4)	295 (89.4)
Yes	2 (3.8)	26 (11.6)	35 (10.6)
χ^2 (P-value)		2.87 (P = 0.24)	
**Previous diagnosis of high TG** [b]			
No	50 (94.3)	206 (92.0)	301 (91.2)
Yes	3 (5.7)	18 (8.0)	29 (8.8)
χ^2 (P-value)		0.61 (P = 0.74)	

[a] Data are presented as mean ± SD. Differences in measurements between groups were analyzed using student's t-test.

* denotes significantly different than no intake group (P ≤0.05)

** denotes significantly different than no intake group (P ≤0.01)

[b] Data are presented as n (n%). $\chi 2$ is the Chi-square test or Fisher's Exact test value followed by its P-value.

BP, blood pressure; TC, total cholesterol; TG, triglycerides

Fat percentage was measured using a portable calibrated scale.
